# The Parkinson’s disease *DJ-1*/*PARK7* gene controls peripheral neuronal excitability and painful neuropathy

**DOI:** 10.1093/brain/awae341

**Published:** 2024-11-02

**Authors:** Sang Hoon Lee, Raquel Tonello, Kihwan Lee, Jueun Roh, Arthur Silveira Prudente, Yong Ho Kim, Chul-Kyu Park, Temugin Berta

**Affiliations:** Pain Research Center, Department of Anesthesiology, University of Cincinnati Medical Center, Cincinnati, OH 45267, USA; Pain Research Center, Department of Anesthesiology, University of Cincinnati Medical Center, Cincinnati, OH 45267, USA; Pain Research Center, Department of Molecular Pathobiology, College of Dentistry, New York, NY 10010, USA; Tooth-Periodontium Complex Medical Research Center, Seoul National University, Seoul 03080, Republic of Korea; Gachon Pain Center and Department of Physiology, College of Medicine, Gachon University, Incheon 21999, Republic of Korea; Pain Research Center, Department of Anesthesiology, University of Cincinnati Medical Center, Cincinnati, OH 45267, USA; Gachon Pain Center and Department of Physiology, College of Medicine, Gachon University, Incheon 21999, Republic of Korea; Gachon Pain Center and Department of Physiology, College of Medicine, Gachon University, Incheon 21999, Republic of Korea; Pain Research Center, Department of Anesthesiology, University of Cincinnati Medical Center, Cincinnati, OH 45267, USA

**Keywords:** Parkinson’s disease, *DJ-1* gene, peripheral neuropathy, oxidative stress, TRPA1 signalling, neuropathic pain

## Abstract

Parkinson’s disease is a progressive neurodegenerative disease with well-documented motor symptoms and less recognized, but significant, non-motor symptoms. These non-motor symptoms include prodromal pain and peripheral neuropathy, the causes of which are unknown.

We investigated the role of *DJ-1*/*PARK7*, a Parkinson’s disease-associated gene, in prodromal pain and peripheral neuropathy. Using *Dj-1*-deficient mice, we conducted comprehensive sensory tests, cutaneous staining, molecular analyses and electrophysiological studies on mouse and human primary sensory neurons from dorsal root ganglia.

We found that these mice exhibited cold hypersensitivity, oxidative stress, and neuropathy of the cutaneous fibres of primary sensory neurons before any motor impairments were observed. Mechanistically, DJ-1 in primary sensory neurons regulated this hypersensitivity and neuropathy via TRPA1 signalling. Interestingly, we discovered that DJ-1 also plays a role in the progression of chemotherapy-induced peripheral neuropathies. Pain and mechanisms associated with these neuropathies were exacerbated in *Dj-1*-deficient mice but were significantly reduced by the pharmacological activation of *Dj-1*. Importantly, we also confirmed the expression of *DJ-1* and its therapeutic potential in human primary sensory neurons.

Thus, we uncover a peripheral mechanism of *DJ-1* and propose that it might serve as a new target for developing therapeutic approaches for Parkinson’s disease-linked and other painful neuropathies.

## Teaser

Parkinson's disease may present with pain and neuropathy. Lee *et al.* show that mice lacking the Parkinson's disease gene *DJ-1* exhibit cold hypersensitivity and neuropathy before showing motor impairments. This effect is mediated through TRPA1 signalling, suggesting DJ-1 as a potential therapeutic target for painful neuropathies.

## Introduction

Parkinson’s disease is a neurodegenerative disease generally characterized by the loss of midbrain dopaminergic neurons and motor symptoms such as tremor, rigidity and bradykinesia.^1^ Parkinson’s disease also presents with several non-motor symptoms, such as sleep disturbances, fatigue, mood changes, autonomic dysfunction and pain.^2^ Remarkably, up to 70% of Parkinson’s disease experience pain, which is often poorly evaluated and inadequately treated.^3,4^ Although this pain is typically attributed to brain dysfunction or muscle rigidity, it often precedes motor impairments and is frequently associated with peripheral sensory neuropathy that is resistant to current treatments aimed at reducing motor symptoms.^5-7^ Studies using quantitative sensory testing show that about one-third of patients with Parkinson’s disease develop painful peripheral neuropathy with progressive alterations of tactile, thermal and other somatosensory sensations, which can precede the onset of motor impairments by several years.^8-11^

Similar to humans, mouse models of Parkinson’s disease have been shown to develop sensory impairments and pain that can precede motor symptoms.^12^ Interestingly, a recent study using a mouse model of Parkinson’s disease, which involves a loss-of-function knockin mutation of PTEN-induced kinase (*Pink1*), in addition to the human A53T mutation of alpha-synuclein (*Scna*), has shown the development of prodromal changes in thermal sensation and peripheral neuropathy.^13^

Numerous genes have been linked to Parkinson’s disease, including those like *PINK1*, which regulate mitochondrial function and oxidative stress.^14^ The deglycase protein DJ-1, encoded by the parkin 7 (*PARK7*) gene, is highly conserved across species, and its inherited and missense mutations have been identified as causes of autosomal recessive forms of Parkinson’s disease.^15^ DJ-1 also acts as a mediator for other genes, such as *PINK1* and *PARK2*, which are associated with autosomal recessive forms of Parkinson’s disease.^16^ Additionally, decreased expression and function of DJ-1 have been observed in human tissues of patients with sporadic Parkinson’s disease.^17-21^ Mechanistically, DJ-1 is a component of the endogenous mechanisms of cellular protection and antioxidant defence induced by oxidative stress in neurodegenerative and inflammatory diseases.^22-27^*Dj-1* knockout (*Dj-1*^−/−^) mice show some motor impairments, nigrostriatal dopaminergic deficits and increased neuronal sensitivity to toxic damage.^28-32^ Although the literature presents conflicting views^33,34^ on the extent to which these mice replicate all features of Parkinson’s disease and their progression, they are frequently used as a model to investigate the underlying mechanisms of motor impairments associated with the condition.^32,35-37^ However, the sensory symptoms and their underlying mechanisms in *Dj-1*^−/−^ mice remain unexplored. Investigating these aspects could unveil new therapeutic strategies for addressing pain and peripheral neuropathy associated with Parkinson’s disease and other neuropathic pain conditions.

In this study, we used *Dj-1*^−/−^ mice in conjunction with motor and sensory behavioural testing, peripheral neuropathy assessments and electrophysiology to define both the aetiology and the mechanisms of Parkinson’s disease pain and peripheral sensory neuropathy. Our findings indicate a peripheral role of DJ-1 in pain and sensory neuropathy through its expression and regulation of transient receptor potential ankyrin 1 (TRPA1) in dorsal root ganglion (DRG) primary neurons. Furthermore, we demonstrate the therapeutic and translational potential of targeting DJ-1 in Parkinson’s disease and in chemotherapy-induced peripheral sensory neuropathies.

## Materials and methods

### Study approvals and animals

Human DRG tissues were collected from a deidentified patient, and all procedures were approved by the Institutional Review Boards of Gachon University and Samsung Medical Center. All animal procedures and experiments received approval from the Institutional Animal Care and Use Committee at the University of Cincinnati and Gachon University. They were also conducted in accordance with the National Institute of Health’s *Guide for the Care and Use of Laboratory Animals*. CD1 mice (Cat. No. 22] were used for pharmacological and biochemical studies and purchased from Charles River Laboratories. The following mouse lines were obtained from the Jackson Laboratory: C57BL/6J control mice (Cat. No. 000664), *Dj-1*^−/−^ mice (Cat. No. 006577), *Trpa1*^−/−^ mice (Cat. No. 006401) and *Calca^cre/EGFP^* (CGRP-EGFP, Cat. No. 033168). *Dj-1*^−/−^*Trpa1*^−/−^ mice were generated by crossing *Dj-1*^−/−^ mice with *Trpa1^−/−^* mice. Male and female mice aged 8–16 weeks were used for the open field test, whereas all other behavioural, electrophysiological and biochemical studies used 8-week-old mice. Mice were housed four per cage at 22°C ± 0.5°C under a controlled 14 h–10 h light–dark cycle, with food and water available *ad libitum*. Sample sizes were determined based on our previous similar studies.^38,39^ All investigators were blind to animal phenotypes and treatments.

### Reagents and drug delivery

Allyl isothiocyanate (AITC, Cat. No. 377430), paclitaxel (PAX, Cat. No. T7402), vincristine (Cat. No. V8388), HC030031 (Cat. No. H4415), methylglyoxal (Cat. No. M0252) and kaempferol 3-*O*-β-rutinoside (K3OβR, Cat. No. 90242) were purchased from MilliporeSigma. DJ-1-mimicking peptide ND-13 (i.e. YGRKKRRKGAEEMETVIPVD) and scrambled control (YGRKKRRDVPIVTEMEEAGK) peptide were synthetized by Biomatik, whereas the TAT-FITC peptide (Cat. No. AS-27042) was purchased from Anaspec. Small interfering RNAs (siRNAs) targeting DJ-1 (siDJ-1, Cat. No. s81228) and non-targeting control (siCTRL, Cat. No. 4390844) were obtained from Thermo Fisher Scientific. Primers for quantitative real-time RT-PCR were also obtained from Thermo Fisher Scientific. Their sequences are listed in Supplementary Table 1. To enhance the uptake by DRG cells, we diluted the siRNAs (3 µg) with 10 µl of solution containing 5% glucose and 2.62 µl of in vivo-jetPEI® (Polyplus, Cat. No. 201-10G) before intrathecal delivery, as previously described.^38,39^ Drugs were delivered by intraplantar or intrathecal injections. Intrathecal injections by spinal puncture were used to deliver reagent into CSF and DRG tissues. A valid spinal puncture was confirmed by a reflexive tail flick after the needle entry into the subarachnoid space.

### Statistics

Statistical analysis was performed with Prism v.10.0 (GraphPad). All the data are expressed as the mean ± standard error of the mean (SEM). Biochemical, immunohistochemical and behavioural data were analysed using Student’s unpaired *t*-test (two groups) and one-way or two-way ANOVA followed by a *post hoc* test specified in the figure legends. Statistical details for each experiment are provided in Supplementary Table 2. The criterion for statistical significance was *P* < 0.05. Illustrator v.25.0 (Adobe) was used for illustrations and figure organization.

Details of all experimental procedures are described in the Supplementary material.

## Results

### *Dj-1* knockout mice exhibit prodromal cold hypersensitivity

**Figure 1:**
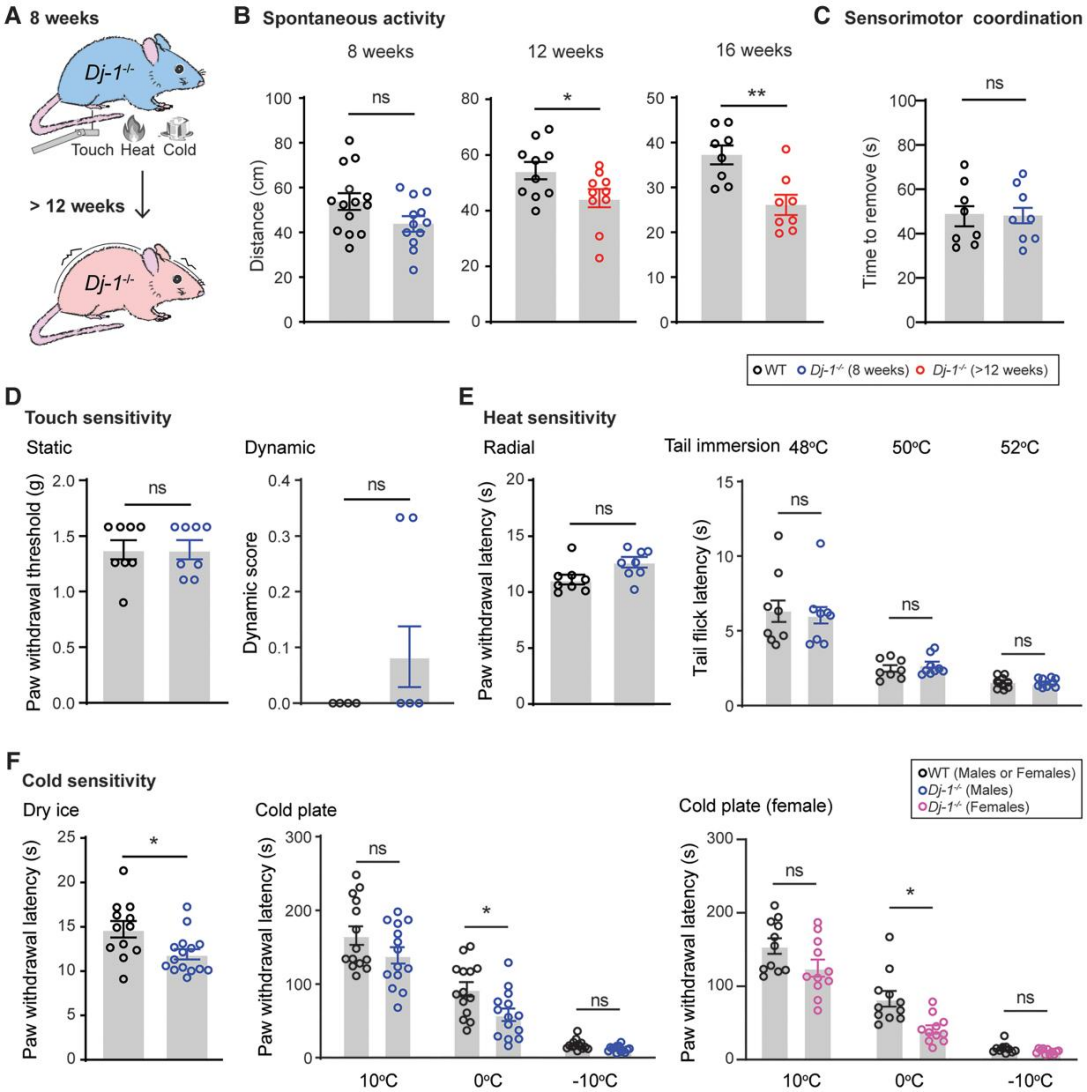
***Dj-1* global knockout (*Dj-1*^−/−^) mice exhibit prodromal cold hypersensitivity.** (**A**) Schematic illustration of *Dj-1*^−/−^ mouse time line for motor impairment and prodromal sensory testing. *Dj-1*^−/−^ and wild-type (WT) mice were assessed at 8 weeks of age for: (**B**) spontaneous activity using the open-field test (*n* = 8–14 male and female mice per group); (**C**) sensorimotor coordination using the adhesive removal test (*n* = 8 male mice per group); (**D**) mechanical sensitivity in response to stimuli by von Frey filament (static, *n* = 8 male mice per group, *left*) and brush (dynamic, *n* = 4 or 5 male mice per group, *right*); (**E**) heat sensitivity using the Hargreaves test (radial, *n* = 10 male mice per group, *left*) and the hot water tail immersion test (*n* = 8 male mice per group, *right*); and (**F**) cold sensitivity using the dry ice test (*n* = 12 male mice per group, *left*) and cold plate test (*n* = 14 male mice per group, *n* = 11 female mice per group). The two-tailed unpaired *t*-test was used for **B**–**D**, the *left* graph of **E**, and the *left* graph for the males in **F**. Two-way ANOVA followed by Šídák’s multiple comparisons test was used for **E***right*, the *right* graph for the males in **F**, and the females in **F**. Error bars indicate the mean ± SEM; *P < 0.05 and **P < 0.01; ns = not significant.

**Figure 2:**
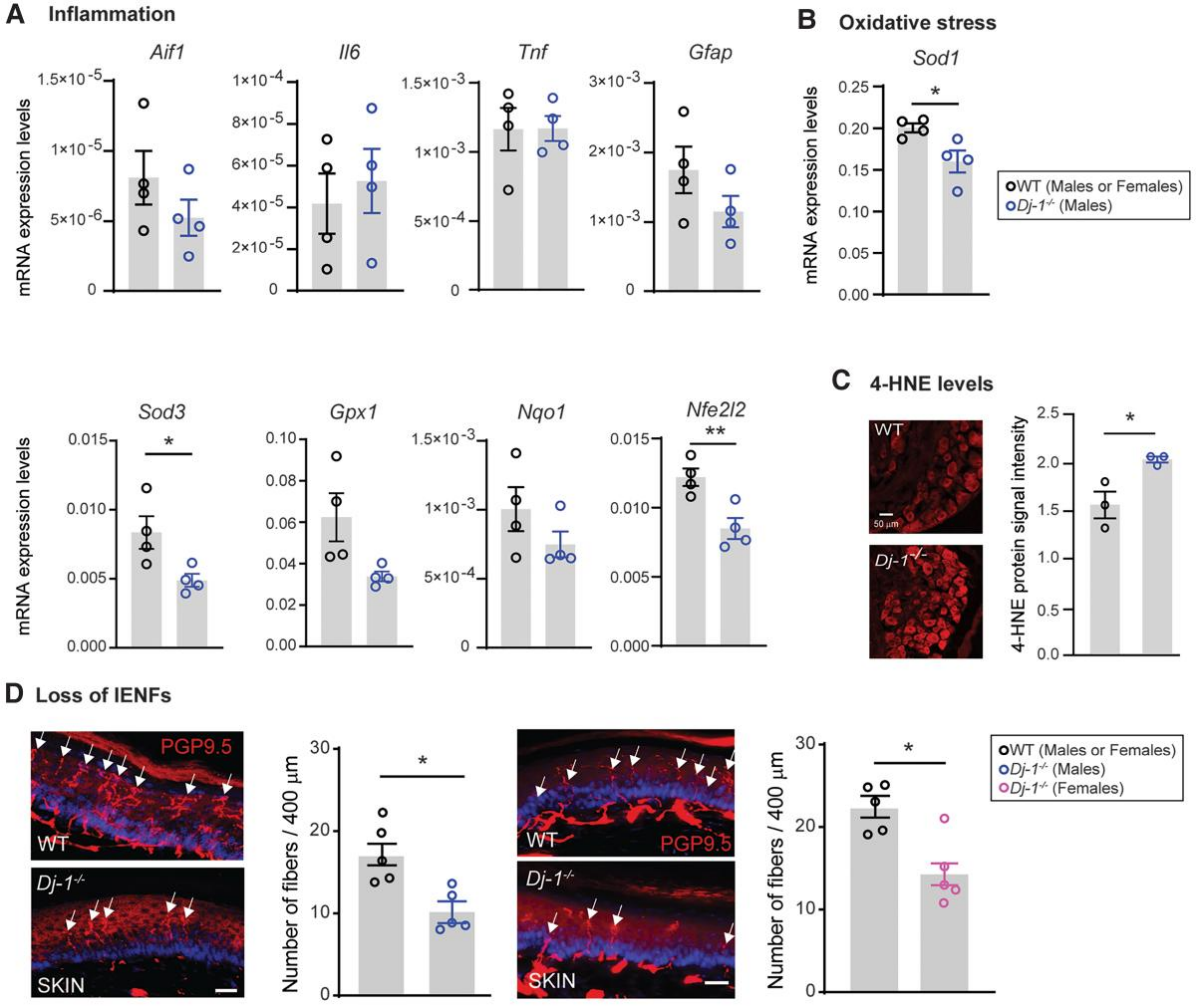
**Lack of DJ-1 expression leads to increased oxidative stress in dorsal root ganglion tissue and peripheral neuropathy.** (**A** and **B**) Expression in wild-type (WT) and *Dj-1*^−/−^ mice (*n* = 4 male mice per group) of gene markers for inflammation (**A**) and genes associated with antioxidant defence and DJ-1 (**B**). (**C**) Representative immunofluorescent images (*left*) and quantification (*right*) of 4-hydroxynonenal (4-HNE) expression in dorsal root ganglion tissues of WT and *Dj-1*^−/−^ mice (*n* = 3 male mice per group). (**D**) Representative immunofluorescence images (*left* in both male and female) and quantification (*right* in both male and female) for the PGP9.5^+^ peripheral nerve fibres in the skin of WT and *Dj-1*^−/−^ mice (scale bar = 20 µm, *n* = 5 male mice per group). Statistical analysis was by two-tailed unpaired *t*-test (**B**–**E**). Error bars indicate the mean ± SEM; *P < 0.05 and **P < 0.01.

Most Parkinson’s disease research has focused on motor impairments and brain-specific mechanisms, with little attention given to the non-motor symptoms and potential contributions of the peripheral nervous system. However, data from single-cell RNA sequencing reveal that several genes associated with Parkinson’s disease are expressed in both mouse and human peripheral sensory neurons (Supplementary Fig. 1A–C). This suggests a potential link between Parkinson’s disease and the commonly reported symptoms of pain and peripheral neuropathies.^5-7^ Notably, DJ-1 (encoded by the *Park7* gene) is highly expressed in these peripheral sensory neurons (Supplementary Fig. 1B). *Dj-1* global knockout (i.e. *Dj-1*^−/−^) mice have long been used as a Parkinson’s disease animal model.^12^ Yet, it remains unclear whether prodromal pain and peripheral neuropathy are present in *Dj-1*^−/−^ mice (Fig. 1A). These mice exhibit nigrostriatal dopaminergic deficits and progressive motor impairments, including reduced open-field locomotion and rotarod fall-off latencies.^32,35-37^ However, debate exists in the literature regarding the extent to which these mice replicate all features of Parkinson’s disease and, in particular, the progression of their motor impairments.^34^ Thus, we initially characterized the time course of the previously reported reduced open-field locomotion in these knockout mice compared with wild-type control mice^32^ and confirmed a progressive deficit in spontaneous activity starting in 12-week-old *Dj-1*^−/−^ mice (Fig. 1B). Importantly, no deficit in spontaneous activity, sensorimotor coordination and forced motor activity (i.e. rotarod fall-off latencies) were observed in 8-week-old *Dj-1*^−/−^ male and female mice (Fig. 1B and C andSupplementary Fig. 2A and B).

Therefore, we sought to determine whether any prodromal sensory impairments were present in 8-week-old *Dj-1*^−/−^ mice by examining their sensory responses to a range of mechanical and thermal stimuli (Fig. 1A). There were no differences between *Dj-1*^−/−^ and wild-type male mice in responses to static and dynamic mechanical stimuli (Fig. 1D) or in thresholds to noxious heat and tail immersion in hot water (Fig. 1E). Likewise, *Dj-1*^−/−^ female mice showed intact responses to mechanical and heat stimuli (Supplementary Fig. 2C and D). However, *Dj-1*^−/−^ male mice displayed a significant increased cold sensitivity when dry ice was applied to the glabrous skin of their hindpaw, and both male and female mice presented cold hypersensitivity when exposed to 0°C in the cold plate test (Fig. 1F). This cold hypersensitivity was also confirmed in male and female mice in responses to evaporative cooling evoked by application of acetone (Supplementary Fig. 2E).

To rule out the possibility that cold hypersensitivity results from developmental impairments of sensory neurons in *Dj-1*^−/−^ mice, we characterized these neurons molecularly and anatomically. These mice exhibited normal central innervations of the primary afferents labelled by calcitonin gene-related peptide (CGRP) and isolectin (IB4) in the spinal dorsal horn (Supplementary Fig. 3A) and normal expression patterns of the neurochemical markers neurofilament high (NF200), CGRP and IB4 in DRG tissue (Supplementary Fig. 3B). Transcriptional expression also confirmed the normal expression of additional markers associated with various subpopulations of DRG neurons (Supplementary Fig. 3C). We conclude that DJ-1 deficiency causes cold hypersensitivity, which is not attributable to developmental impairments in sensory neurons.

### *Dj-1* knockout mice exhibit prodromal peripheral neuropathy

Peripheral neuropathy, although arising from diverse causes, often presents with similar symptoms and mechanisms, including inflammation and oxidative stress in DRG tissue, along with intraepidermal nerve fibre (IENF) loss in the skin.^40^ Transcriptional analyses of genes related to inflammation revealed no significant changes in DRG tissue from *Dj-1*^−/−^ mice when compared with wild-type mice (Fig. 2A). However, genes related to DJ-1 and antioxidative defences,^41^ such as *Sod1* and *Nfe2l2*, were significantly decreased in *Dj-1*^−/−^ mice compared with wild-type mice (Fig. 2B). In line with an oxidative stress imbalance and vulnerability, the staining 4-hydroxynonenal (4-HNE) protein adduct, a well-known marker for oxidative stress,^42^ was increased in DRG neurons from *Dj-1*^−/−^ mice (Fig. 2C). This vulnerability to oxidative stress was also evident in cultured DRG neurons from *Dj-1*^−/−^ mice. These neurons had a larger increase in 4-HNE and dihydroethidium staining, another oxidative stress marker,^43^ compared with wild-type mice when exposed to hydrogen peroxide and rotenone (Supplementary Fig. 4A–C). We also observed impaired neurite outgrowth in cultured DRG neurons from *Dj-1*^−/−^ mice (Supplementary Fig. 4D). This indicates an increased vulnerability to oxidative stress, because proper mitochondrial function is required for energy production in this process.^44^

Additionally, immunofluorescent staining with the protein gene product 9.5 (PGP9.5), a pan-neuronal marker widely used to assess peripheral neuropathy,^45^ in the paw glabrous skin revealed a significant decrease in IENF in both male and female *Dj-1*^−/−^ mice compared with wild-type mice (Fig. 2D). It is noteworthy that the transcriptional expression of PGP9.5 (encoded by the gene *Uchl1*) is not altered in DRG tissue (Supplementary Fig. 3C), supporting a loss of these fibres instead of a loss of PGP9.5 expression. Altogether, this observation, along with increased oxidative stress, indicates the presence of prodromal peripheral sensory neuropathy in 8-week-old *Dj-1*^−/−^ mice.

### DJ-1 expression in DRG neurons controls cold sensitivity and neuropathy via TRPA1

**Figure 3:**
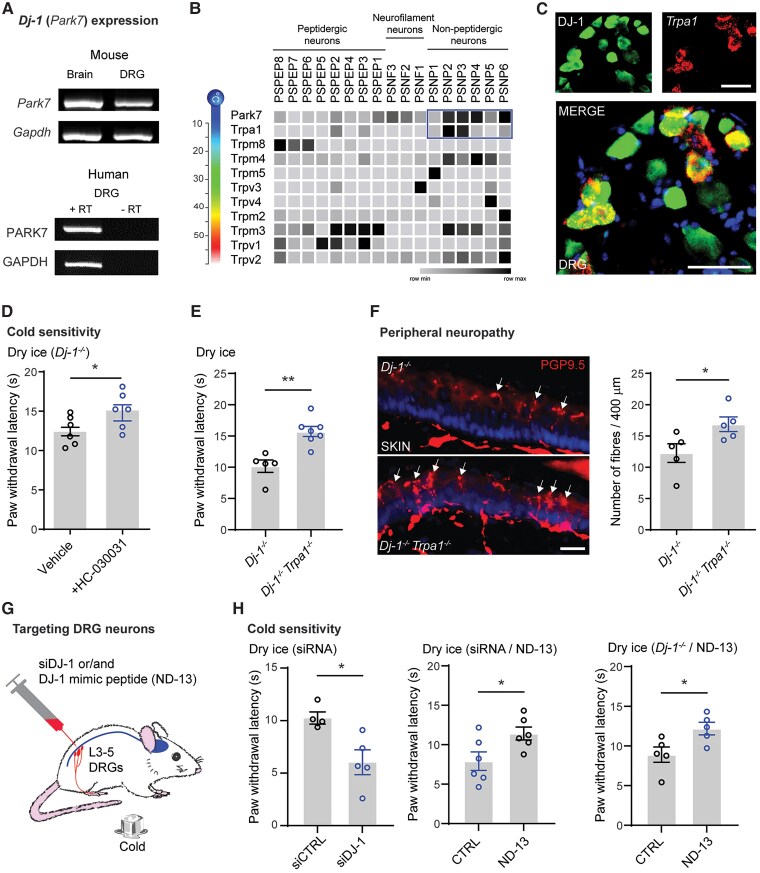
**DJ-1 expression in TRPA1-nociceptors and regulation of cold hypersensitivity and peripheral neuropathy.** (**A**) RT-PCR of *DJ-1* mRNA brain and dorsal root ganglion (DRG) tissues from mouse, as well as human DRG tissue. RT = reverse transcriptase. (**B**) Heat map of DJ-1 and various TRP channel mRNAs in different populations of DRG neurons based single-cell RNA-sequencing data previously published by Zeisel *at al*.^46^ (**C**) Representative images for DJ-1 immunofluorescence and *Trpa1* RNAscope in DRG neurons (scale bar = 50 µm). Dry ice-induced cold sensitivity in: (**D**) *Dj-1*^−/−^ mice 30 min after administration of vehicle or TRPA1 antagonist HC030031 (10 μg/10 μl, intraplantar, *n* = 6 male mice per group); and (**E**) in *Dj-1*^−/−^ and *Dj-1*^−/−^*Trpa1^−/−^* mice (*n* = 5–7 male mice per group). (**F**) Representative immunofluorescence images (*left*) and quantification (*right*) for the PGP9.5^+^ peripheral nerve fibres in the skin of *Dj-1*^−/−^ and *Dj-1*^−/−^*Trpa1*^−/−^ mice (scale bar = 20 µm, *n* = 5 male mice per group). (**G**) Schematic illustration of intrathecal injections of small interfering (si)RNA and peptide targeting DRG tissues. (**H**) Dry ice-induced cold sensitivity in wild-type mice 2 days after injection of siRNA: control (siCTRL) or DJ-1 (siDJ-1) siRNA injection (*n* = 4–5 male mice per group, *left*); siDJ-1-administrated wild-type mice 1 h after control (CTRL) or DJ-1-mimicking peptide (ND-13) injections (*n* = 6 male mice per group, *middle*); *Dj-1*^−/−^ mice 1 h after control (CTRL) or DJ-1-mimicking peptide (ND-13) injections (*n* = 6 male mice per group, *right*). Statistical analysis: two-tailed unpaired *t*-test (**D**–**F** and **H**). Error bars indicate the mean ± SEM; *P < 0.05 and **P < 0.01.

To extend the above observations and investigate the underlying mechanisms further, we characterized the expression of DJ-1 in DRG tissue. Notably, DJ-1 mRNA is expressed in both human and mouse DRG tissue (Fig. 3A). Using an antibody validated in *Dj-1*^−/−^ mice (Supplementary Fig. 5A), we determined that DJ-1 protein in mice is mostly expressed in large-sized NF200^+^ and non-peptidergic IB4^+^ DRG neurons (Supplementary Fig. 5B). Previously published single-cell RNA sequencing data by Zeisel *et al*.^46^ confirmed this expression in non-peptidergic DRG neurons enriched in the expression of channels detecting mild to hot temperature,^47^ including TRPM3, TRPM4 and TRPV2 (Fig. 3B). Relevant to our study, these data also show DJ-1 expression in DRG neurons that express the cold-sensing channel TRPA1, but not TRPM8 (Fig. 3B). Notably, ∼80% of DRG neurons expressing TRPA1 mRNA also expressed DJ-1 protein, whereas ∼60% of DJ-1^+^ neurons expressed TRPA1 mRNA (Fig. 3C and Supplementary Fig. 5C and D). TRPA1 could be associated with specific phenotypes observed in *Dj-1*^−/−^ mice, because it is activated by cold stimuli and modulated by oxidative stress adducts, such as 4-HNE, which are known to contribute to painful peripheral neuropathy.^48,49^ Indeed, we demonstrated that inhibiting TRPA1 with HC030031, a widely used and highly specific TRPA1 channel blocker,^50,51^ reduced the cold hypersensitivity found in *Dj-1*^−/−^ mice when dry ice was applied (Fig. 3D). The development of this hypersensitivity in *Dj-1*^−/−^ mice was prevented in double-knockout *Dj-1*^−/−^*Trpa1*^−/−^ mice (Fig. 3E). To assess the role of TRPA1 in the DJ-1 phenotype, we examined *Trpa1*^−/−^ mice alone. These mice showed no change in cold sensitivity compared with wild-type mice (Supplementary Fig. 6A), aligning with reports of the minor role of TRPA1 in acute cold sensation without reactive oxygen species or pro-inflammatory activators.^52,53^ Importantly, the decrease in IENF observed in *Dj-1*^−/−^ mice compared with wild-type mice (Fig. 2D) was also prevented in double-knockout *Dj-1*^−/−^*Trpa1*^−/−^ mice (Fig. 3F).

To assess the contribution of DJ-1 in DRG neurons to cold hypersensitivity and TRPA1 activity, we used intrathecal injections of siRNA (i.e. siDJ-1) to reduce DJ-1 expression in wild-type mice and a cell-permeable mimicking peptide (i.e. ND-13^54^) to rescue DJ-1 function in siDJ-1-treated mice or in *Dj-1*^−/−^ mice (Fig. 3G). We demonstrated that both the siRNA and cell-permeable peptide can target the DRG tissue specifically, leaving the spinal tissues untouched (Supplementary Fig. 6B–D). More importantly, we showed that treatment with siDJ-1 increased the cold sensitivity of mice to dry ice application, whereas ND-13 reduced it compared with the respective control mice (Fig. 3H). Remarkably, the rescue of DJ-1 function in DRG by ND-13 was sufficient to reverse the cold hypersensitivity found in *Dj-1*^−/−^ mice (Fig. 3H), suggesting peripheral therapeutic potential for Parkinson’s disease-linked non-motor symptoms.

Furthermore, we tested whether DJ-1 modulates TRPA1 activity using the specific TRPA1 agonist allyl isothiocyanate (AITC, i.e. mustard oil).^55^ Cultured DRG neurons from *Dj-1*^−/−^ mice exhibited stronger calcium responses to AITC than those from wild-type mice (Supplementary Fig. 6E). Previous studies have also shown that AITC induces strong spontaneous nocifensive behaviours, such as paw lifting, shaking and licking, following its intraplantar injection.^50,56^ In accordance with our *in vitro* findings on TRPA1 modulation by DJ-1, mice treated with siDJ-1 exhibited increased spontaneous nocifensive behaviours after AITC injection, whereas mice treated with ND-13 displayed reduced responses in comparison to their respective control mice (Supplementary Fig. 6F). In summary, these data indicate that DJ-1 is linked to TRPA1 expression and activity, controlling both the development of cold hypersensitivity and peripheral neuropathy.

### DJ-1 expression controls the progression of painful peripheral neuropathy

**Figure 4:**
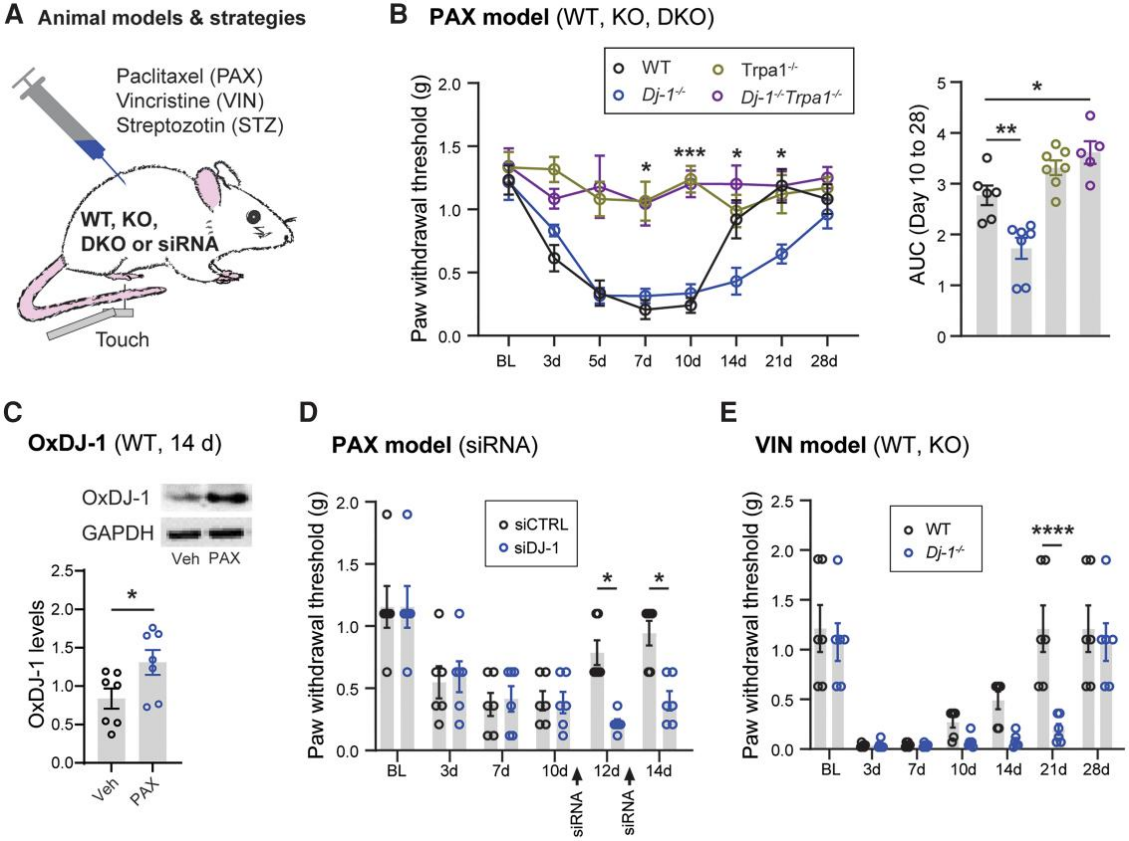
**DJ-1 controls the progression of painful peripheral neuropathy.** (**A**) Schematic illustration of animal models and strategies. (**B**) Progression of mechanical hypersensitivity induced by paclitaxel (PAX) in wild-type (WT), *Dj-1* global knockout (*Dj-1*^−/−^), *Trpa1* global knockout (*Trpa1*^−/−^) or *Dj-1*^−/−^*Trpa1*^−/−^ mice (*n* = 5–7 mice per group). Asterisks indicate a comparison between *Dj-1*^−/−^ and *Dj-1*^−/−^*Trpa1*^−/−^ mice. Area under the curve (AUC) from Fig. 2B, *left* during the resolution of PAX-induced mechanical hypersensitivity (from Day 10 to Day 28, *n* = 5–7 male mice per group). (**C**) Expression of oxidized DJ-1 (OxDJ-1) in dorsal root ganglion of WT mice 14 days after the first injection of paclitaxel (*n* = 7 male mice per group). (**D**) Resolution of PAX-induced mechanical hypersensitivity is also impaired in mice intrathecally injected with siDJ-1 (2 µg/10 µl) compared with siCTRL, on Day 10 and Day 12 (*n* = 6 male mice per group). (**E**) Progression of mechanical hypersensitivity induced by vincristine in WT and *Dj-1*^−/−^ mice (*n* = 6 male mice per group). Statistical analysis: two-tailed unpaired *t*-test (**C**), one-way ANOVA followed by Dunnett’s multiple comparisons test (**B**, *right*), two-way ANOVA followed by Tukey’s multiple comparisons test (**B**, *left*) and Šídák’s multiple comparisons test (**D** and **E**). Error bars indicate mean ± SEM; *P < 0.05, **P < 0.01, ***P < 0.001 and ****P < 0.0001.

We also investigated whether DJ-1 could play a role in other types of peripheral neuropathy. We used knockout mice, siRNA injections and animal models of peripheral neuropathies (Fig. 4A). In particular, we used the paclitaxel-induced animal model, because this chemotherapeutic agent is widely recognized to cause mechanical hypersensitivity owing to oxidative stress and TRPA1-dependent neuropathy.^48,57^ Systemic injections of paclitaxel (PAX, 2 mg/kg, two injections administered every other day) induced prolonged mechanical hypersensitivity in wild-type mice, which subsided after 14 days.^58,59^ Notably, this hypersensitivity was not observed in mice lacking TRPA1 (i.e. *Trpa1*^−/−^) expression. However, in *Dj-1*^−/−^ mice, it developed normally but persisted until Day 28, suggesting that DJ-1 plays a role in the resolution of mechanical hypersensitivity (Fig. 4B).

The oxidation of the C106 residue of DJ-1, caused by oxidative stress, is essential for its protective functions.^60^ Consistent with the protective role of DJ-1, we noted a significant increase in the expression levels of this residue (i.e. OxDJ-1) 14 days after PAX, when mechanical hypersensitivity is resolving (Fig. 4C). Furthermore, the knockdown of DJ-1 expression in DRG tissues using siDJ-1 injections starting 10 days after PAX also demonstrated the impairment of mechanical hypersensitivity resolution (Fig. 4D). It is noteworthy that the impairment in resolution of mechanical hypersensitivity also occurred in another mouse model of peripheral neuropathy, this one induced by the chemotherapeutic agent vincristine (Fig. 4E).

Painful peripheral neuropathy is also common in diabetic patients.^61^ Initially, we attempted to model diabetic painful peripheral neuropathy using streptozotocin in mice, as previously reported.^62,63^ However, in our experiments, these mice did not develop hyperglycaemia (data not shown). Therefore, we examined the role of DJ-1 in pain-like behaviours induced by an intraplanar injection of methylglyoxal. Methylglyoxal is generated during the hyperglycaemic states in both type 1 and 2 diabetic patients and might contribute to pain development in diabetic neuropathy.^64,65^ Methylglyoxal injection to *Dj-1*^−/−^ mice, compared with control wild-type mice, resulted in exaggerated nocifensive behaviours and greater mechanical hypersensitivity (Supplementary Fig. 7A and B), supporting a potential role of DJ-1 in diabetic pain. Overall, these data indicate that DJ-1 expression is required for the proper response to the noxious injection of methylglyoxal and resolution of chemotherapy-induced mechanical hypersensitivity.

### DJ-1 is a translational therapeutic target for the treatment of painful peripheral neuropathy

**Figure 5:**
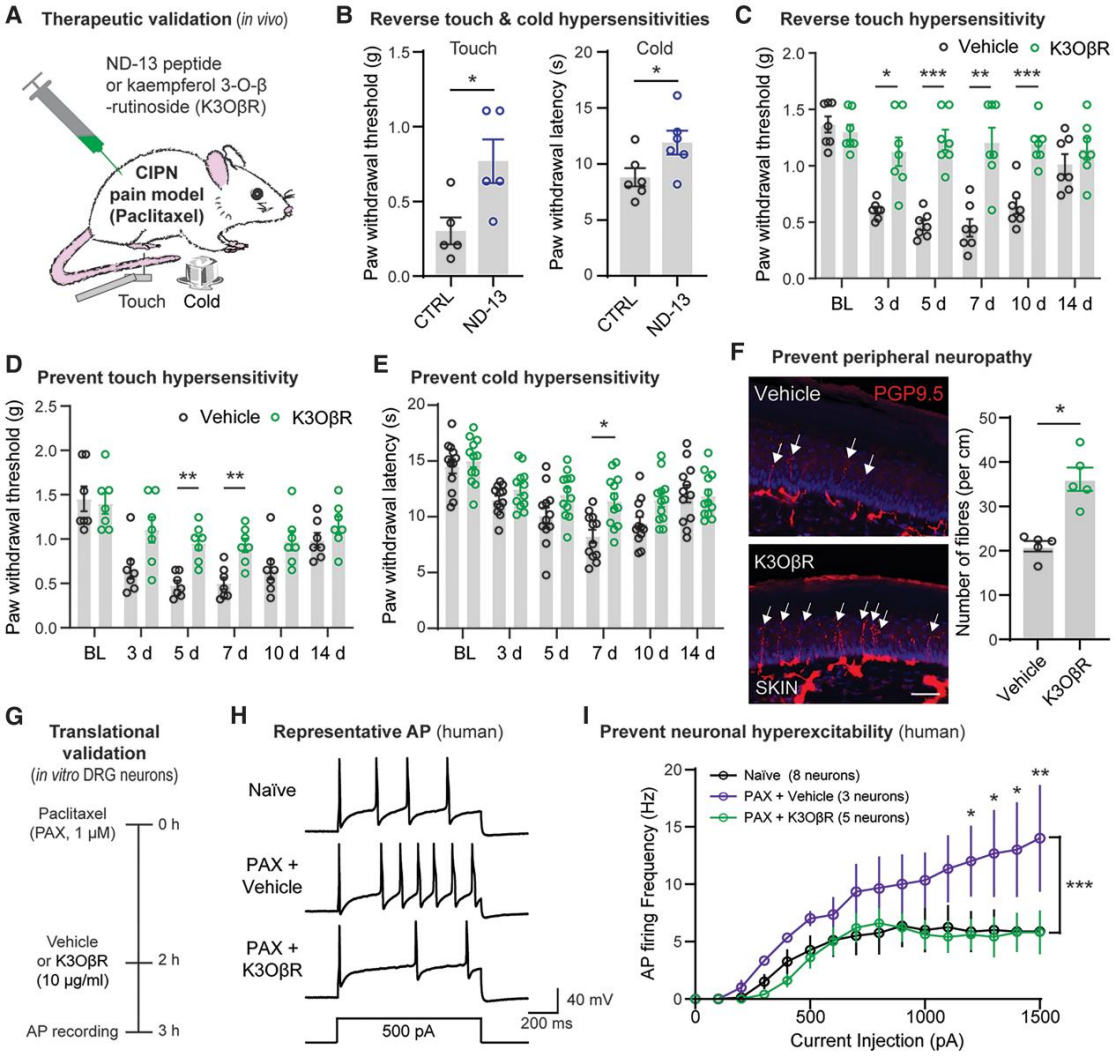
**Targeting DJ-1 alleviates symptoms and mechanisms associated with chemotherapy-induced peripheral neuropathy (CIPN).** (**A**) Schematic illustration of CIPN model induced by paclitaxel (PAX) injections and *in vivo* therapeutic validation of DJ-1-mimicking peptide (ND-13) and kaempferol 3-*O*-β-rutinoside (K3OβR). (**B**) Intrathecal injection of ND-13 (1 μg/10 μl), when compared with a control peptide (CTRL), reversed mechanical and cold hypersensitivities induced by PAX (7 after first PAX injection, *n* = 6 male mice per group). (**C**) PAX-induced mechanical hypersensitivity is reversed 1 h after daily injections of K3OβR (10 μg/10 μl), when compared with a control vehicle (*n* = 7 male mice per group). PAX-induced mechanical (**D**) and cold (**E**) hypersensitivities, which were tested before daily injections, demonstrated a partial prevention by K3OβR (10 μg/10 μl) compared with a vehicle control (*n* = 7 male mice per group). (**F**) Representative immunofluorescence images (scale bar = 20 µm, *left*) and quantification (*right*) for the PGP9.5^+^ peripheral nerve fibres in the skin of mice treated with K3OβR or vehicle for 6 days (intrathecal injections, 10 μg/10 μl, *n* = 5 male mice per group). (**G**) Schematic illustration of protocol used to test the therapeutic effect of K3OβR on PAX-induced hyperexcitability in dorsal root ganglion (DRG) neurons. (**H** and **I**) Representative current-clamp recordings of action potential (AP) traces (**H**) and quantification of AP firing frequencies (**I**) in human DRG neurons in naïve conditions, in addition to those treated with PAX (1 µM) and either vehicle or K3OβR (10 μg/10 μl). Statistical analysis: two-tailed unpaired *t*-test (**B** and **F**), two-way ANOVA followed by Šídák’s multiple comparisons test (**C–E**) and Tukey’s multiple comparisons test (**I**). Error bars indicate the mean ± SEM; *P < 0.05, **P < 0.01 and ***P < 0.001.

We have shown that DJ-1 is expressed across various species and plays a significant role in progression of peripheral neuropathy. This condition remains a challenge in the medical field, because there are currently no US Food and Drug Administration-approved treatments. Recognizing this unmet need, we investigated targeting DJ-1 as a novel therapeutic approach using two promising compounds: ND-13 and K3OβR. As previously mentioned, ND-13 is a cell-permeable peptide designed to mimic DJ-1 function,^54^ whereas K3OβR is a natural flavonoid known for its ability to activate DJ-1 specifically.^66^ We tested the effects of these compounds on paclitaxel (PAX)-induced mechanical and cold hypersensitivity, key symptoms of chemotherapy-induced peripheral neuropathy (Fig. 5A). We found that a single intrathecal application of ND-13 produced a reversal of both mechanical and cold hypersensitivity 7 days after PAX injections (Fig. 5B). The effect of ND-13 lasted only a few hours (data not shown), probably owing to its inclusion of the HIV-derived TAT sequence.^54^ This sequence enables cell membrane penetration and DJ-1 targeting in the cytosol, but ultimately transfers the peptide to the nucleus,^67^ neutralizing its effect. In contrast, repeated intrathecal application of K3OβR (Supplementary Fig. 8A) effectively reversed PAX-induced mechanical and cold hypersensitivities without exhibiting signs of tolerance (Fig. 5C and Supplementary Fig. 8B). It also partly prevented the development of both mechanical hypersensitivity and, to a lesser extent, cold hypersensitivities (Fig. 5D and E). However, K3OβR failed to reverse these mechanical and cold hypersensitivities in *Dj-1*^−/−^ mice (Supplementary Fig. 8C and D), thus validating its specificity towards the activation of DJ-1.

Supporting its therapeutic value in peripheral neuropathy, K3OβR not only prevented PAX-induced symptoms but also mitigated some of its underlying mechanisms. Mice treated with K3OβR showed increased IENF density in skin and decreased 4-HNE levels in DRG tissue in comparison to mice treated with a control vehicle (Fig. 5F and Supplementary Fig. 8E).

It is also well known that PAX promptly activates and increases the number of evoked action potentials (APs) in mouse and in human nociceptors *in vitro*, providing an essential translational evaluation method for new therapeutic targets.^68^ We then examined the APs triggered by current injections through an intracellular electrode in small-sized DRG neurons (i.e. nociceptors; Supplementary Fig. 9A). Neurons from both wild-type and *Dj-1*^−/−^ mice and from a human organ donor were compared in three conditions: naïve, treated with PAX + vehicle, or treated with PAX + K3OβR (Fig. 5G). PAX consistently increased the AP firing in wild-type nociceptors in vehicle-treated neurons. This effect was reversed in nociceptors treated with K3OβR exhibiting similar AP firing to naïve neurons (Supplementary Fig. 9B and C). However, K3OβR failed to reverse the increased AP firing in *Dj-1*^−/−^ mice (Supplementary Fig. 9D and E). Remarkably, K3OβR also reversed the increased AP firing evoked by PAX in human nociceptors (Fig. 5H and I), implying a translational therapeutic value of targeting DJ-1 in DRG neurons.

## Discussion

Pain, a common issue in Parkinson’s disease, is often overlooked and inadequately treated.^3^ This is partly owing to our limited understanding of the mechanisms behind it. In this study, we identify the expression of Parkinson’s disease-related DJ-1 in nociceptors and its role in prodromal pain and peripheral neuropathy through TRPA1 modulation. This research suggests a possible mechanism for peripheral neuropathies in Parkinson’s disease patients and offers new prognostic approaches. Additionally, we reveal the potential of targeting DJ-1 for therapeutic interventions in patients with Parkinson’s disease and other peripheral neuropathies.

Parkinson’s disease, generally characterized by the loss of dopaminergic neurons and motor impairments, is often also accompanied by prodromal pain and sensory neuropathy.^9,10^ The cause of these prodromal symptoms is unclear, but they suggest a particular vulnerability of the peripheral sensory neurons. Despite this, few peripheral sensory neurons in the DRG are dopaminergic, and these symptoms often do not respond to L-DOPA treatment.^6,69^ However, our mining of recently published single-cell RNA sequencing data^46,70^ has revealed the expression of several genes associated with Parkinson’s disease in these peripheral sensory neurons, including *Pink1*, *Scna* and *Park7*. A recent study suggests that some of these genes indeed play a peripheral role in the prodromal symptoms of Parkinson’s disease, showing early changes in thermal sensation and peripheral neuropathy in a Parkinson’s disease mouse model lacking *Pink1* and with A53T *Snca* mutations.^13^ Here, we characterize DJ-1 (encoded by the *Park7* gene) as a new player controlling pain and neuropathy through its actions in peripheral sensory neurons.

The first notable finding of this study is the significant presence of prodromal cold hypersensitivity and peripheral neuropathy in 8-week-old *Dj-1*^−/−^ mice, detected prior to the development of any motor impairments. These mice presented no alteration to tactile or heat stimuli but displayed a profoundly increased sensitivity to all tested cold stimuli, such as dry ice and a cold plate. Of note, *Dj-1*^−/−^ mice showed no gross molecular or anatomical defects, making it unlikely that the observed behaviours are results of developmental defects. However, they exhibited clear signs of peripheral neuropathy, which was indicated not only by the loss of IENF in the skin, but also by the increased vulnerability to oxidative stress and impaired neurite outgrowth in cultured DRG neurons. We found transcriptional decreases in DRGs of genes associated with DJ-1 and cellular antioxidative defences, such as nuclear factor erythroid 2-related factor 2 (NRF2, encoded by the *Nfe2l2* gene). DJ-1 helps to stabilize and activate NRF2,^71^ a crucial transcription factor that manages oxidative stress by transcribing >200 antioxidant genes.^72^ It is plausible that the lack of DJ-1 might cause the destabilization and diminished expression of NRF2, potentially leading to oxidative stress and generation of the 4-HNE observed in *Dj-1*^−/−^ mice. Interestingly, NRF2 has been linked to peripheral neuropathy and proposed as a therapeutic target for the treatment of peripheral neuropathic pain.^73,74^ However, it is possible that the increased oxidative stress in *Dj-1*^−/−^ mice could be NRF2 independent, because DJ-1 can directly regulate mitochondrial functions and activation of multiple antioxidants, such as glutathione and superoxide dismutase.^16,75^ Further investigation is required to test this possibility.

As a multifunctional protein, DJ-1 can be expressed in multiple cell types and play a role in various diseases, including inflammatory diseases.^23^ However, we found that DJ-1 transcripts are prevalent in DRG neurons, in line with the single-cell RNA sequencing database of this tissue,^46,70^ and that it was expressed particularly in neurons expressing the TRPA1 channel, a widely researched transduction molecule in pain research.^55,76^ TRPA1 is found in multimodal nociceptors that are sensitive to noxious cold,^77^ piquant natural substances from mustard and allium plants,^78^ and oxidative stress products.^53^ The role of TRPA1 as a cold nociceptor is still a subject of debate. However, a recent study provided evidence that TRPA1 might function as a noxious cold sensor through cold-induced oxidative stress products, stimulating neurons without directly detecting cold.^49^ In this regard, we observed specifically an increase in the oxidative product 4-HNE in *Dj-1*^−/−^ mice, which is known to sensitize TRPA1 directly, leading to neuronal hyperexcitability and pain.^79^ Additional evidence linking DJ-1 to TRPA1 in nociceptors includes the observations of increased neuronal activity induced by the TRAP1 agonist AITC in cultured nociceptors from *Dj-1*^−/−^ mice compared with wild-type mice. In line with this, AITC also induced increased nocifensive behaviours in knockdown mice injected with siDJ-1, whereas mice treated with the active DJ-1-mimicking peptide ND-13 exhibited reduced nocifensive behaviours. Furthermore, the cold hypersensitivity observed in *Dj-1*^−/−^ mice was reversed in mice treated with the specific TRPA1 inhibitor HC-030031 or in mice lacking TRPA1. Peripheral neuropathy in *Dj-1*^−/−^ mice was also reversed in mice lacking TRPA1. Thus, it is tempting to speculate that TRPA1 could provide a mechanistic framework for improved treatment of prodromal pain and peripheral neuropathy in Parkinson’s disease, which is currently limited owing, in part, to a lack of understanding of its underlying mechanisms.

Cold hypersensitivity and oxidative stress are prominent features of chemotherapy-induced neuropathies, for which there is no US Food and Drug Administration-approved treatment.^80^ Thus, we hypothesized that DJ-1 might play a significant role in these neuropathies and could serve as a therapeutic target. We demonstrated the role of DJ-1 in the resolution of mechanical hypersensitivity induced by the chemotherapeutic drugs paclitaxel and vincristine. We also identified a potential role of DJ-1 in diabetic pain through our experiment with methylglyoxal, a compound generated during hyperglycaemic states in both type 1 and type 2 diabetic patients.^64,65^ Methylglyoxal injection in *Dj-1*^−/−^ mice led to increased nocifensive behaviours and mechanical hypersensitivity compared with wild-type controls, suggesting the involvement of DJ-1 in diabetic pain. However, further research using various animal models is necessary to confirm its potential role in diabetic neuropathies. It is important to note that the role of DJ-1 in mechanical hypersensitivity emerges only when *Dj-1*^−/−^ mice face additional stressors, such as chemotherapy. Increased oxidative stress and TRPA1 activation are likely to drive this effect. Although the exact mechanisms of TRPA1 remain unclear, our findings and prior studies^57,81,82^ indicate its involvement in mechanical hypersensitivity in various peripheral neuropathies. In all models, mechanical hypersensitivity lasted longer in *Dj-1*^−/−^ mice than in wild-type mice after exposure to these drugs. A plausible explanation is that DJ-1 is typically activated only during oxidative stress,^60^ suggesting that DJ-1 contributes to these neuropathies only when there is enough accumulation of oxidative stress. However, we also found that *Dj-1*^−/−^ mice showed exaggerated nocifensive behaviours and greater mechanical hypersensitivity compared with control mice when injected with methylglyoxal. This suggests both acute protective and pro-resolution functions of DJ-1 in various models of painful neuropathies.

Most importantly, we demonstrated that targeting DJ-1 can alleviate pain associated with chemotherapy-induced neuropathy. Initially, we revealed that ND-13, an active DJ-1-mimicking peptide, could reverse paclitaxel-induced mechanical and cold hypersensitivities. This peptide has proved effective in protecting neuronal cultures from the effects of relevant neurotoxins in conditions such as Parkinson’s disease, amyotrophic lateral sclerosis and ischaemic injury.^54,83,84^ However, the HIV TAT sequence in this peptide is a drawback because it can directs it to the nucleus,^67^ losing the necessary cytosolic localization for activity. To address this limitation, we used kaempferol, a natural flavonoid proven to offer cell protection through DJ-1 activation.^66^ Kaempferol is also an appealing treatment option because flavonoids have consistently demonstrated a safe profile in both preclinical and clinical studies.^85^ Notably, we found that kaempferol (i.e. K3OβR) both reversed and partly prevented paclitaxel-induced mechanical and cold hypersensitivities. We also demonstrated the specificity of kaempferol through DJ-1, because its beneficial effects disappeared when administered to *Dj-1*^−/−^ mice. The strong binding of K3OβR to the C106 residue might provide specificity for DJ-1. This is supported by the binding of K3OβR to the wild-type DJ-1 protein, but not to the DJ-1 protein containing a mutation in the C106 residue.^66^ Interestingly, quercetin, structurally similar to kaempferol, binds C106 but acts independently of DJ-1. The additional OH group of kaempferol in its benzene ring might enhance its binding by filling the space between cysteine 106 and histidine 142 in DJ-1, contributing to its activity and specificity.^86,87^ K3OβR prevented not only the development of symptoms, but also the mechanisms associated with peripheral neuropathy, reducing oxidative stress in DRG tissue and IENF loss in the skin. It is important to note that our study focused on evoked pain behaviours. The addition of non-evoked behaviour measures in future studies would provide a more comprehensive assessment of the effectiveness of the proposed treatments in addressing the full spectrum of an animal’s ongoing pain experience.^88^ However, overall, our study suggests that kaempferol could potentially be used as a therapy to reverse and prevent various peripheral sensory neuropathies.

Understanding and targeting DJ-1 has translational value owing to its conservation across species and its implications in both inherited and sporadic Parkinson’s disease.^15,17-21^ But does kaempferol have translational potential for pain treatment? We demonstrated this translational potential using mouse and human cultured DRG neurons exposed *in vitro* to paclitaxel.^68^ We found that paclitaxel enhanced the excitability of small-sized DRG neurons cultured from both mice and humans (i.e. potential nociceptors), as previously reported.^89,90^ However, this increased excitability was reversed when co-treated with kaempferol. Again, we confirmed the specificity of the actions of kaempferol through DJ-1, because this reversal effect was abolished in DRG neurons from *Dj-1*^−/−^ mice. It is important to note that these *in vitro* experiments explore the acute effects of paclitaxel and kaempferol. However, clinical studies indicate acute pain after paclitaxel treatment, the intensity of which can predict severe neuropathy as treatment progresses.^91-93^ Thus, understanding the acute pathophysiology of paclitaxel on mouse and human DRG neurons aids in the discovery and validation of new therapeutics, such as kaempferol.

## Conclusion

In summary, Parkinson’s disease is a neurodegenerative disorder with motor symptoms and non-motor symptoms, including pain.^2^ Here, we presented new data suggesting a peripheral role for Parkinson’s gene *DJ-1* in painful peripheral neuropathies associated with Parkinson’s disease and cancer drugs. We demonstrated its expression in nociceptors and its effect through TRPA1. Importantly, we confirmed the therapeutic potential of kaempferol via DJ-1 for preventing and treating peripheral neuropathies.

## Supplementary Material

awae341_Supplementary_Data

## Data Availability

All raw data used for figure generation in this study are provided in Supplementary Table 3.
