# Supplementary material for: The Parkinson’s disease DJ-1/PARK7 gene controls peripheral neuronal excitability and painful neuropathy
Source: Brain. 2024 Nov 2;148(5):1639–51. doi: 10.1093/brain/awae341 (PMC12073980; doi:10.1093/brain/awae341)
Supplement: awae341_Supplementary_Data [file awae341_supplementary_data.zip › brain-2024-01276-File007.pdf]

Supplementary Figures

Parkinson's gene controls peripheral neuronal excitability and painful neuropathy

Lee et al.

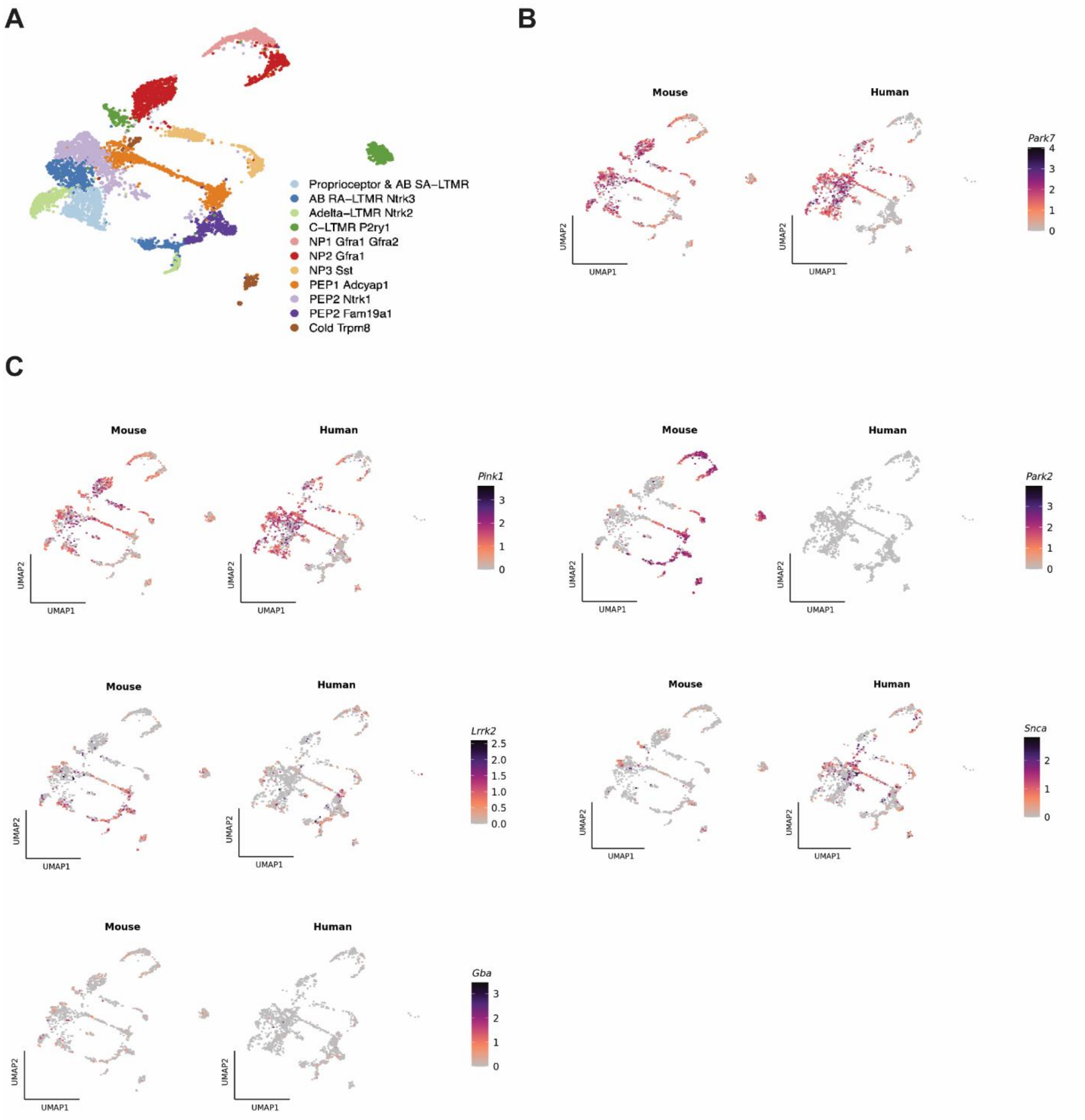

Supplementary Fig. 1. UMAP of Seurat-integrated DRG neurones across mouse and human for genes associated with Parkinson's disease (PD). (**A**) UMAP colored by DRG neuronal subtypes. UMAPs for the expression of *Park7* (i.e. DJ-1) (**B**), and other PD's genes **(C)** *Pink1*,

Park2, *Lrrk2*, *Scna* and *Gba* in mouse and human DRG neurones based on online single-cell RNA-seq data (http://research-pub.gene.com/XSpeciesDRGAtlas/#).

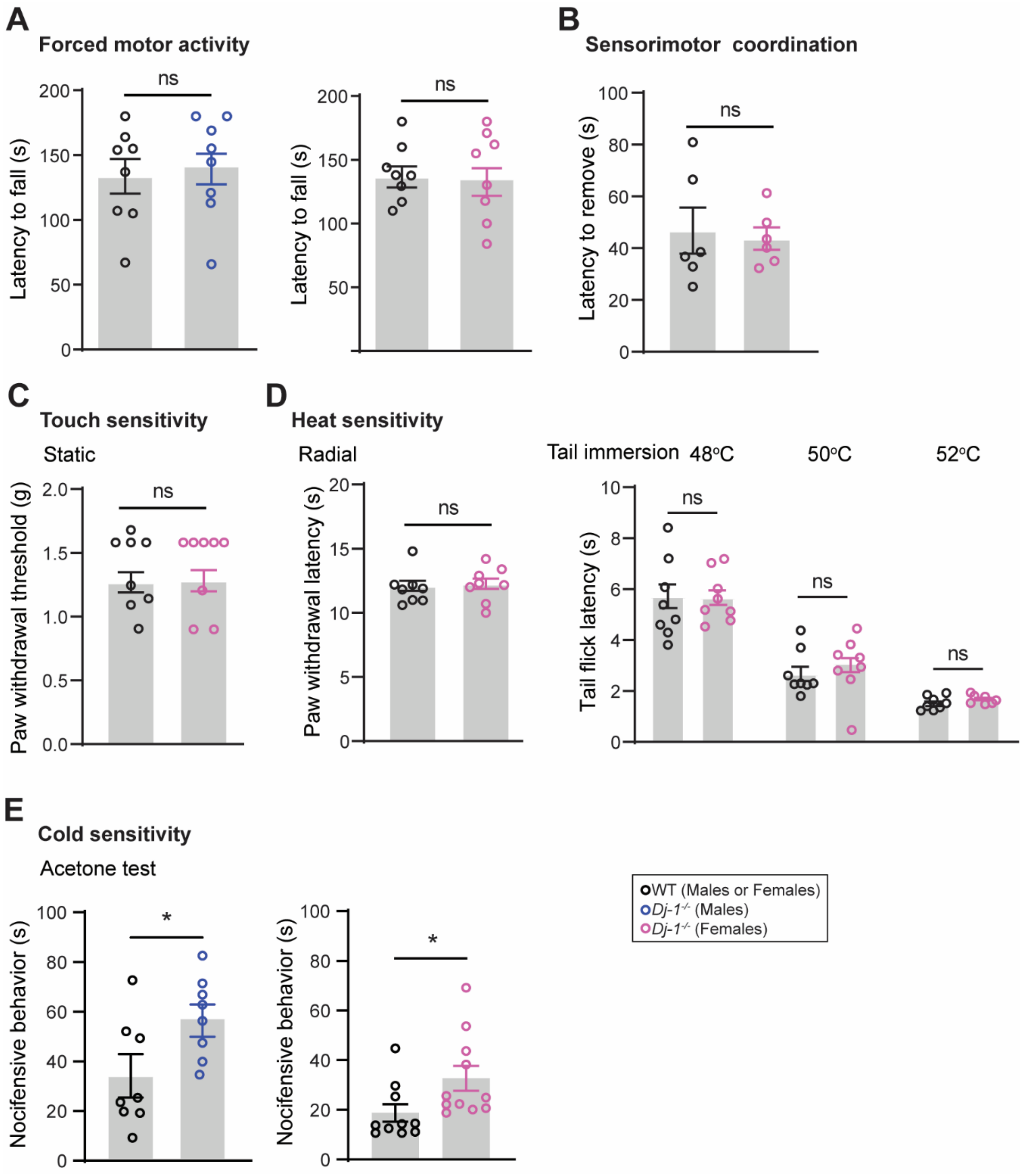

Supplementary Fig. 2. Motor and sensory behavioral assessment in male and female DJ-1 global knockout (*Dj-1*$^{-/-}$) mice. (**A**) Forced activity of male (left) and female (right) *Dj-1*$^{-/-}$ and wild-type (WT) mice at 8 weeks of age measured using the rotarod test (n = 8 mice/group). (**B**) Sensorimotor coordination of female WT and *Dj-1*$^{-/-}$ mice was assessed using the adhesive removal test (n = 6 female mice/group). (**C**) Mechanical sensitivity of female WT and *Dj-1*$^{-/-}$ mice was

measured in response to stimuli by von Frey filament (n = 8 female mice/group). (**D**) Heat sensitivity of female WT and *Dj-1*$^{-/-}$ mice was evaluated using the Hargreaves test (radial, n = 8 female mice/group, left) and the hot water tail immersion test (n = 8 female mice/group, right). (**E**) Cold sensitivity of male (n = 8 mice/group) and female (n = 10-11 mice/group) WT and *Dj-1*$^{-/-}$ mice was evaluated using the acetone test. Statistical analysis: two-tailed unpaired t-test (A, B, C, E and the left side in D), two-way ANOVA followed by Šídák's multiple comparisons test (the right side in D). Error bars indicate mean ± s.e.m. and *$p < 0.05$. ns is not significant.

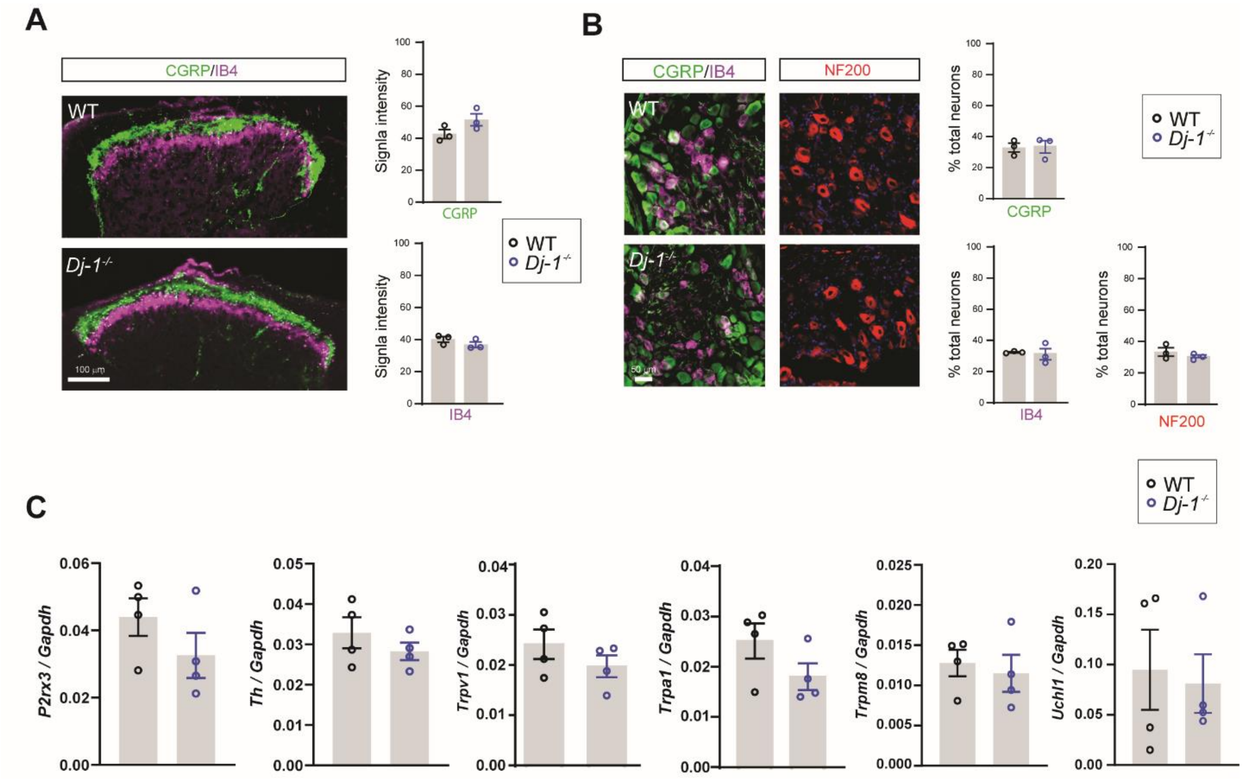

Supplementary Fig. 3. Neuroanatomical and transcriptional characterization of DJ-1 global knockout (*Dj-1*$^{-/-}$) mice. (**A**) Representative immunofluorescent images (left) and quantifications (right) of the expression of CGRP (lamina I) and IB4 (lamina II) in DRG neurones of wild-type (WT) and *Dj-1*$^{-/-}$ mice (n = 3 male mice/group). (**B**) Representative immunofluorescent images (left) and quantifications (right) of the expression of NF200 (Neurofilament), CGRP (peptidergic neurones), and IB4 (non-peptidergic neurones) in DRG neurones of both WT and *Dj-1*$^{-/-}$ mice (n = 3 male mice/group). Expression in WT and *Dj-1*$^{-/-}$ mice (n = 4 male mice/group) of gene markers for: various subtypes of DRG neurones (**C**). Statistical analysis: two-tailed unpaired t-test (A-C). Error bars indicate mean ± s.e.m.

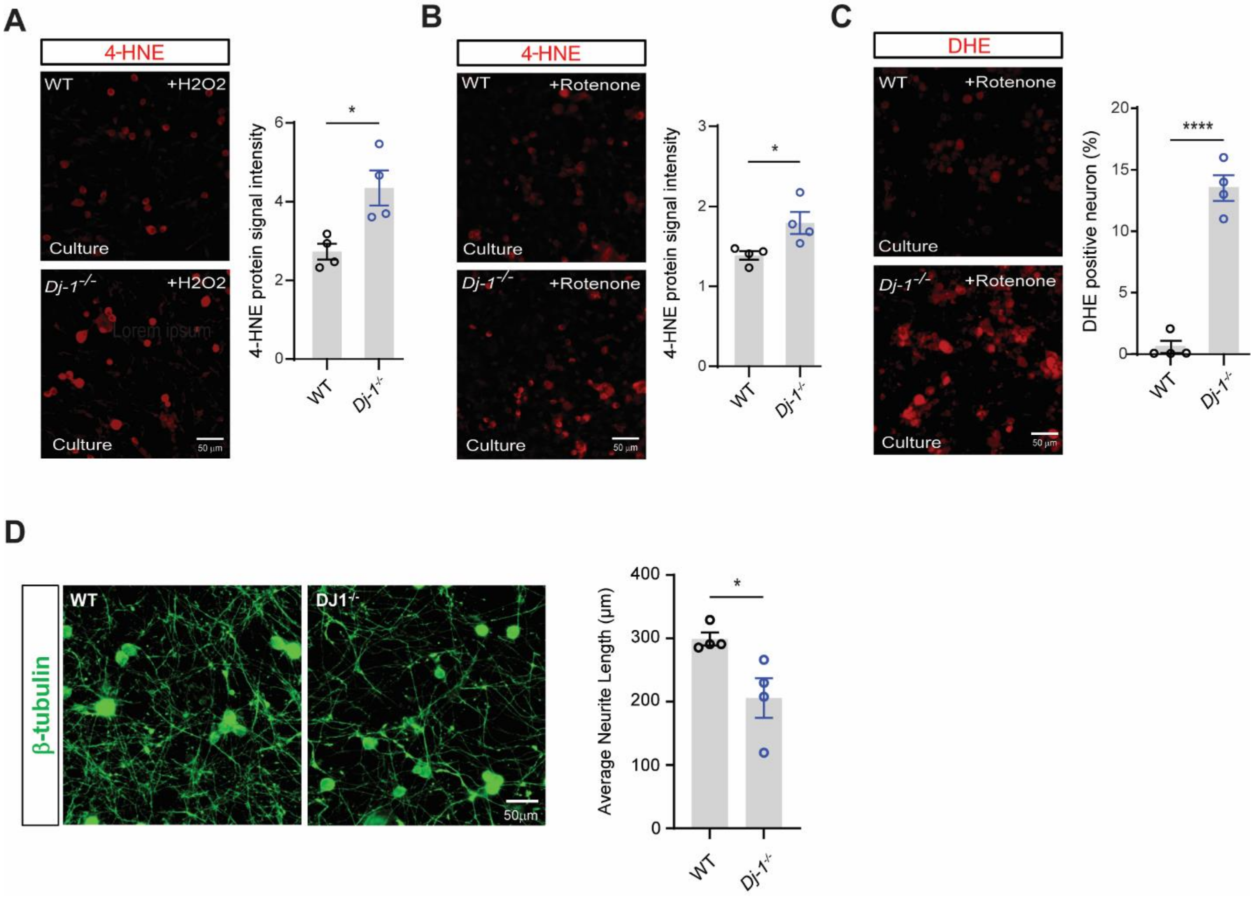

Supplementary Fig. 4. DJ-1 controls oxidative stress and peripheral neuropathy. (**A**) Representative immunofluorescent images (left) and quantification (right) of 4-HNE expression after H2O2 treatments (100mM, 24hr) in cultured DRG neurones from WT and *Dj-1*$^{-/-}$ male mice (n = 4 wells/group). (**B**) Representative immunofluorescent images and quantification of 4-HNE expression after rotenone treatments (2.5mM, 24hr) in cultured DRG neurones of both WT and *Dj-1*$^{-/-}$ male mice (n = 4 wells/group). (**C**) Representative images and quantification of dihydroethidium (DHE) expression after rotenone treatments (2.5mM, 24hr) in cultured DRG neurones of both WT and *Dj-1*$^{-/-}$ male mice (n = 4 wells/group). (D) Representative images and quantification of neurite length in cultured DRG neurones of both WT and *Dj-1*$^{-/-}$ male mice (n = 4 wells/group). Statistical analysis: two-tailed unpaired t-test (A-C). Error bars indicate mean ± s.e.m. and *$p < 0.05$, ****$p < 0.0001$.

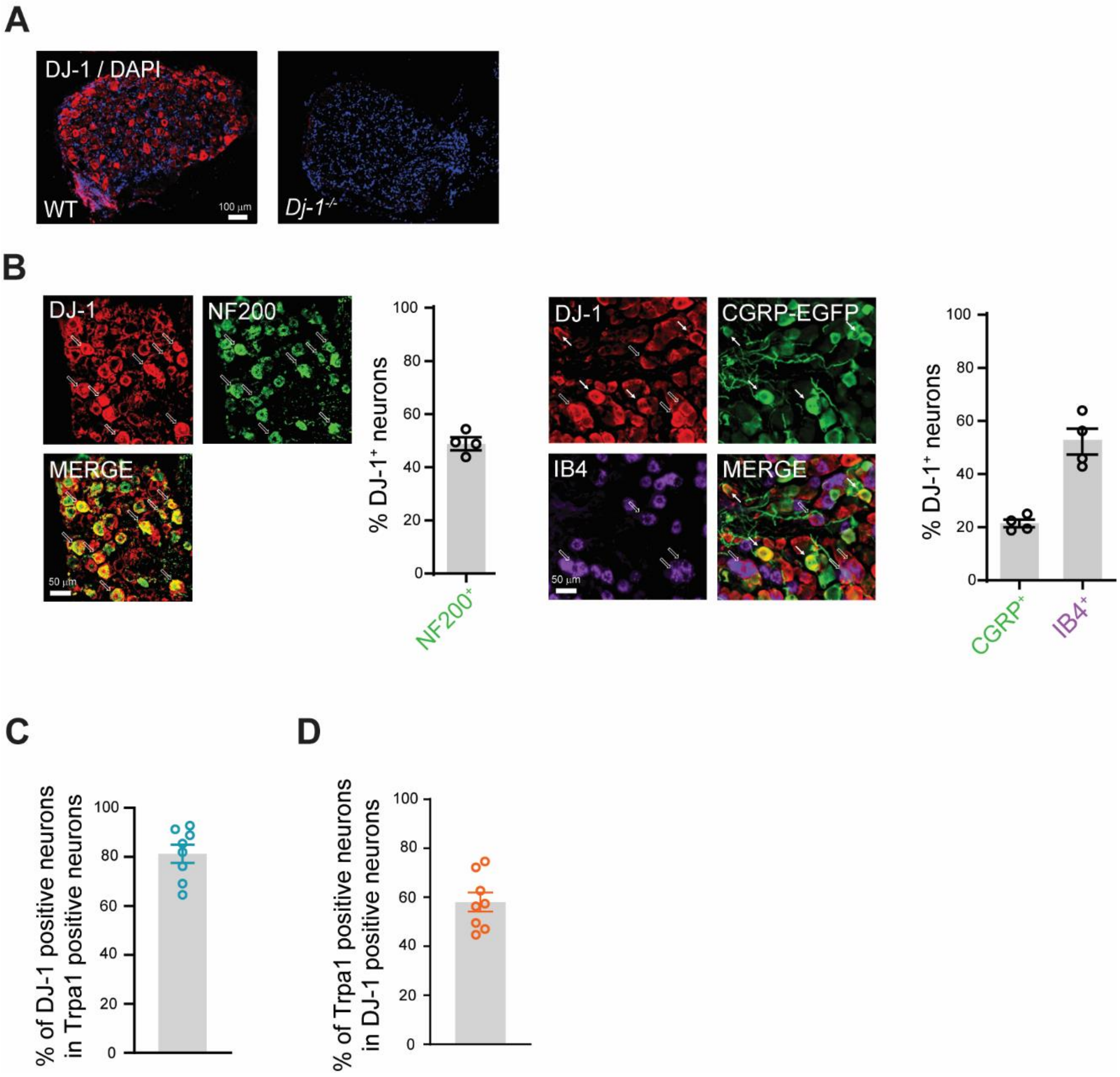

Supplementary Fig. 5. DJ-1 expression in DRG neurones. (**A**) Representative immunofluorescent images of DJ-1 in DRG tissue of wild-type (WT) and DJ-1 global knockout (*Dj-1*$^{-/-}$) male mice. DAPI was used as tissue counterstaining. (**B**) Representative immunofluorescent images (left of graphs) and quantification (right of images) of DJ-1 expression in NF200, CGRP or IB4 expressing DRG neurones (n = 4 DRGs/group). **(C)** quantification of DJ-1 expression in Trpa1 expressing DRG neurones (n = 8 DRGs/group) (D) quantification of Trpa1 expression in DJ-1 expressing DRG neurones (n = 8 DRGs/group). Two male mice were used for quantification.

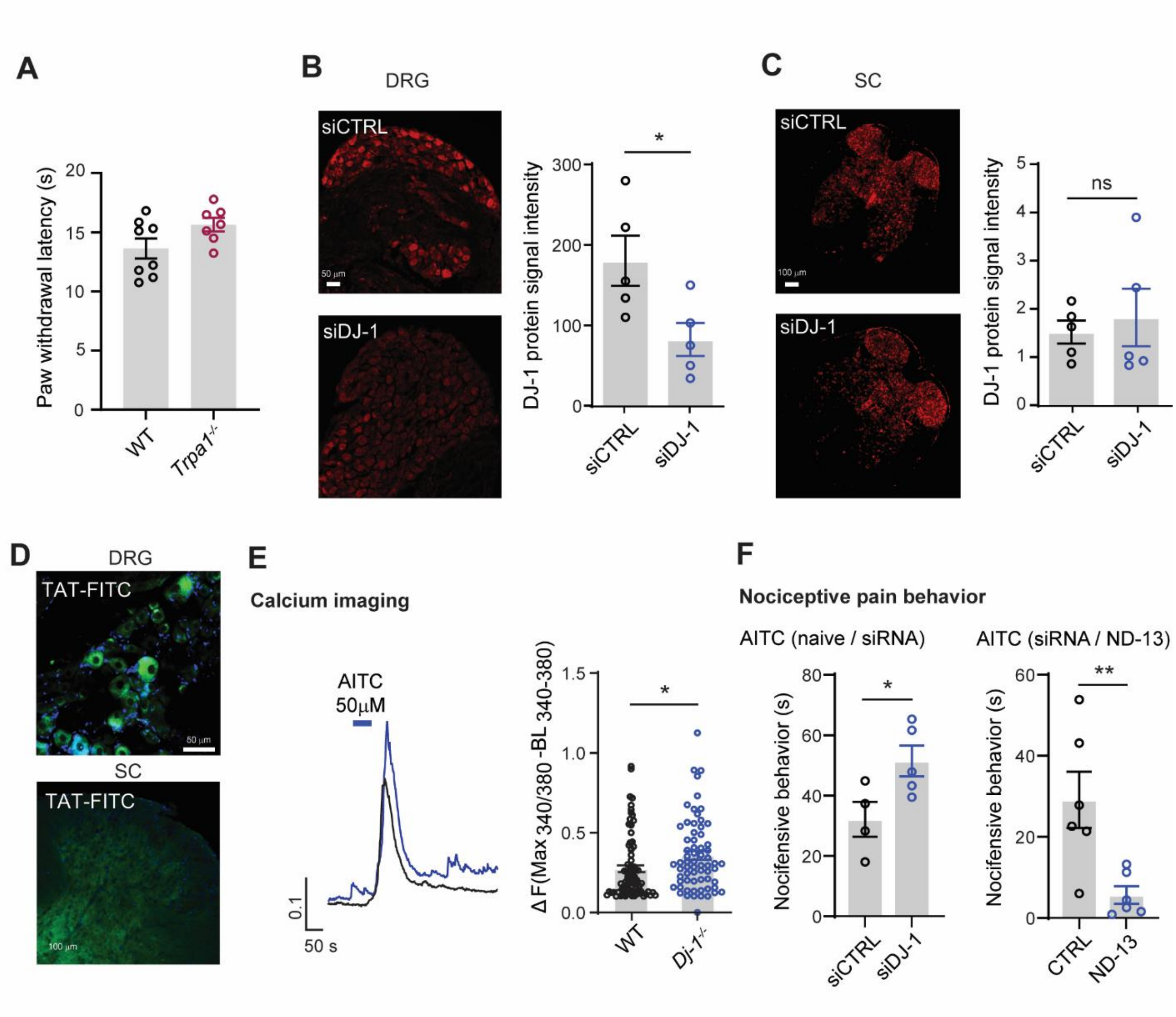

Supplementary Fig. 6. DJ-1 loss- and gain-of-function in DRG neurones controls AITC-induced calcium response and nocifensive behaviors. (A) Dry ice induced cold hypersensitivity in both naïve WT and *Trpa1*$^{-/-}$ mice (n = 7-8 male mice/group). (**B**) Representative immunofluorescent images (left) and quantification (right) of DJ-1 expression in DRG 2 days after intrathecal injection of siCTRL or siDJ-1 (2μg/10μl, n = 5 male mice/group). (**C**) Representative immunofluorescent images (left) and quantification (right) of DJ-1 expression in spinal cord (SC) tissue after s intrathecal injection of siCTRL or siDJ-1 (n = 5 male mice/group). (**D**) Representative images of TAT-FITC peptide in DRG and SC. TAT-FITC after intrathecal injection. (**E**) Representative trace (left) and quantification (right) of calcium response in DRG neurones of wild-type (WT) and DJ-1 global knockout (*Dj-1*$^{-/-}$) mice following AITC treatment (n = 66-84 neurones/group). Three male mice were used for quantification. (**F**) AITC (intraplantar,

200nmol/20μl)-induced nocifensive pain responses (i.e. time of lifting, shacking, licking) measured in WT mice 2 days after the intrathecal injection of siCTRL or siDJ-1 (n = 4-5 male mice/group, left). AITC-induced nocifensive pain responses in WT mice injected with siDJ-1 1hr after intrathecal injection of control peptide (CTRL) or DJ-1 mimicking peptide (ND-13, 1μg/10μl, n = 6 male mice/group, right). Statistical analysis: two-tailed unpaired t-test (A, B, D, and E). Error bars indicate mean ± s.e.m. and $^{*}p < 0.05$, $^{**}p < 0.01$. ns is not significant.

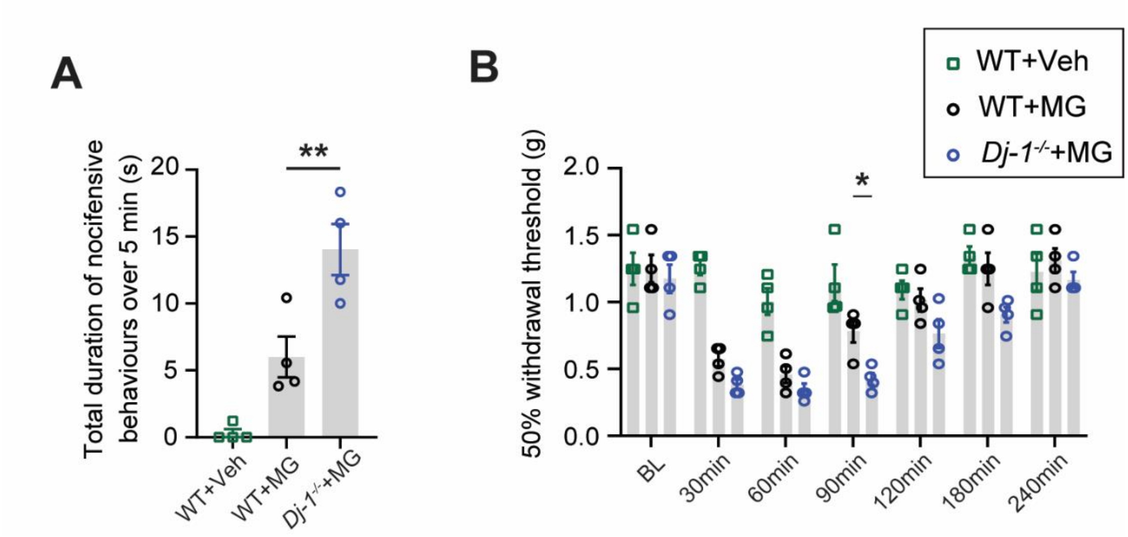

Supplementary Fig. 7. DJ-1 regulates pain responses in diabetes-like models. Intraplanar injection of Methylglyoxal (MG, 30µg/20µl) induced nocifensive behaviours (A) and mechanical hypersensitivity (B) (n = 4 male mice/group). Statistical analysis: one-way ANOVA followed by Tukey's multiple comparisons test (A), and two-way ANOVA followed by Šídák's multiple comparisons test (B). Error bars indicate mean ± s.e.m. and $*p < 0.05$, $**p < 0.01$.

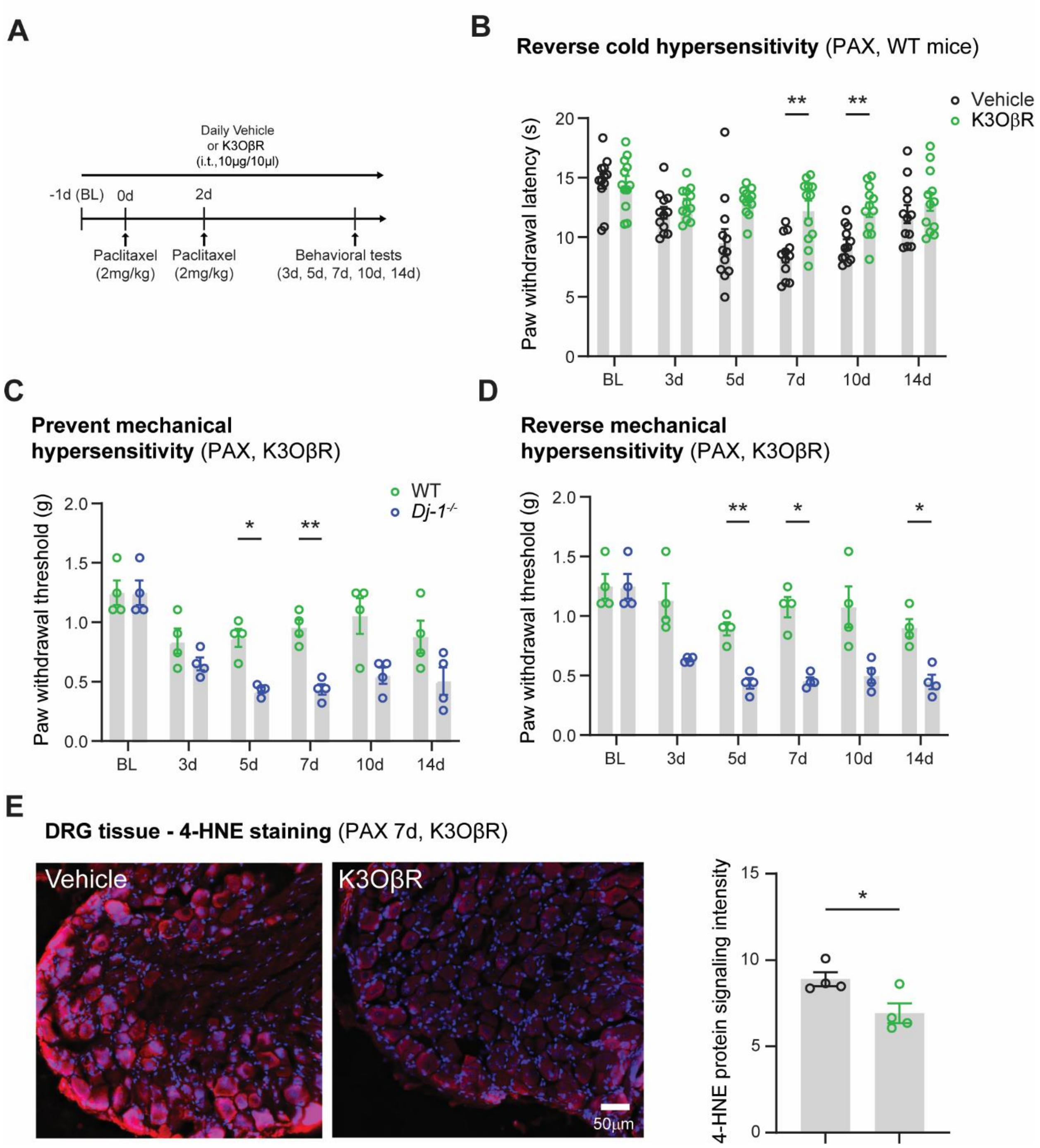

Supplementary Fig. 8. Daily injections of kaempferol (K3OβR) alleviates symptoms and mechanisms of PAX-induced painful peripheral neuropathy. (**A**) Schematic illustration of paclitaxel (PAX)-induced peripheral neuropathy, behavioral testing, and daily treatments. (**B**) PAX-induced cold hypersensitivity is partially reversed 1 hour after daily intrathecal (i.t.) injections of K3OβR, when compared to a control vehicle (n = 7 male mice/group). PAX-induced

mechanical hypersensitivity is neither prevented (**C**) or reversed (**D**) by daily injections of K3OβR in DJ-1 global knockout (*Dj1*$^{-/-}$) mice, compared to wild-type (WT) mice (n = 4 male mice/group). (**E**) PAX-induced oxidative stress (i.e. 4-HNE immunofluorescence) was decreased by daily injections of K3OβR in WT mice (n = 4 male mice/group). Representative images on the left, and quantification on the right. Statistical analysis: two-tailed unpaired t-test (E), two-way ANOVA followed by Šídák's multiple comparisons test (B-D). Error bars indicate mean ± s.e.m. and *$p < 0.05$, **$p < 0.01$.

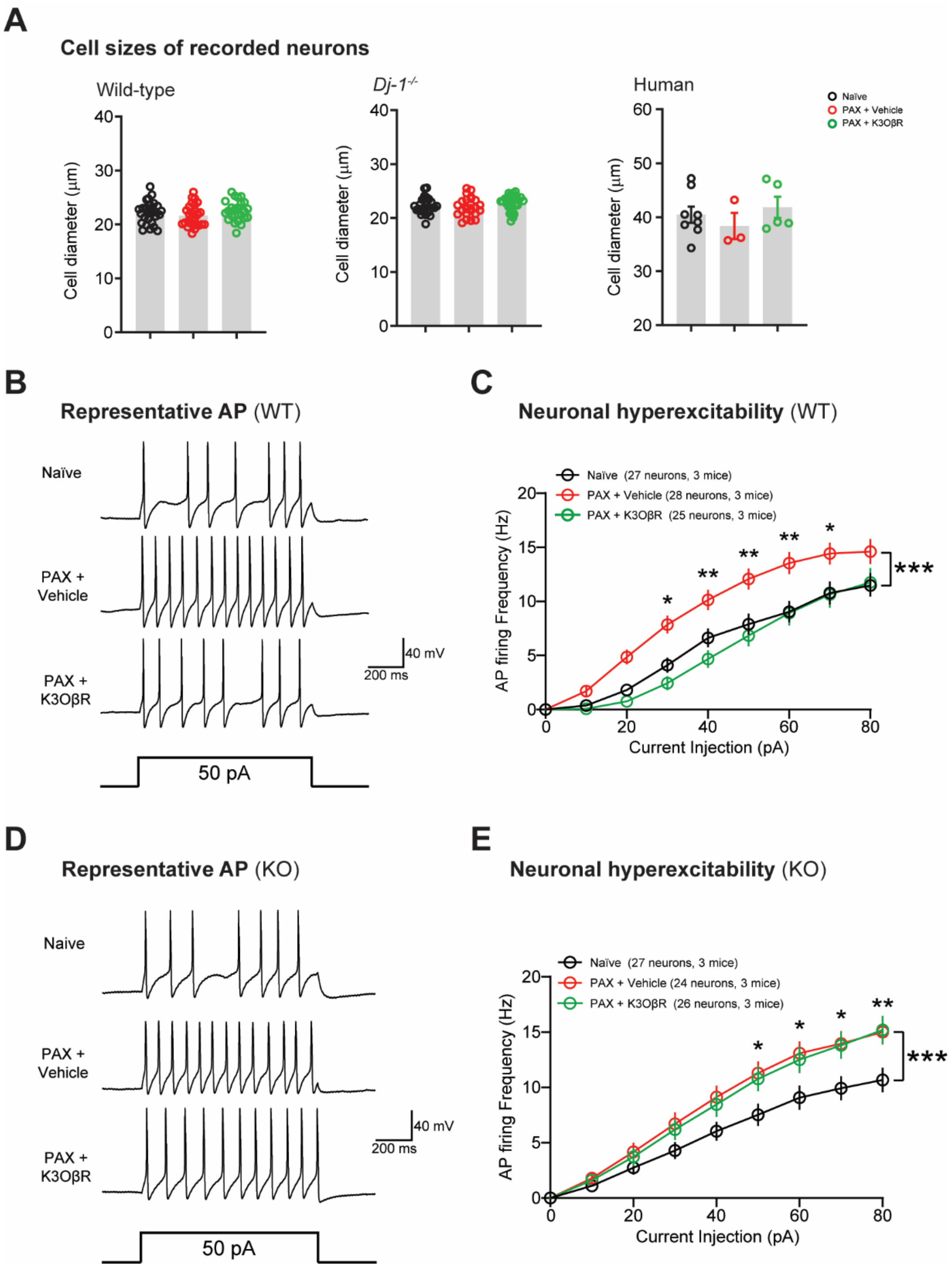

Supplementary Fig. 9. Kaempferol (K3OβR) controls paclitaxel-induced hyperexcitability *in vitro* DRG nociceptors. (**A**) Putative nociceptors were measured on the based of their cell diameters of <25 µm for DRG neurones from wild-type (WT) and DJ-1 global knockout (*Dj-1*$^{-/-}$) mice, and <50 µm for neurones from human. Representative current clamp recordings of action potential (AP) traces (**B**) and quantification of AP firing frequencies (**C**) in DRG neurones from

WT male mice under naïve conditions, as well as those treated with PAX (1 μM) and either vehicle or K3OβR (10μg/10ml). Representative current clamp recordings of AP traces (**D**) and quantification of AP firing frequencies (**E**) in DRG neurones from *Dj-1*$^{-/-}$ male mice under naïve conditions, as well as those treated with PAX (1 μM) and either vehicle or K3OβR (10μg/10ml). Three WT and *Dj-1*$^{-/-}$ male mice were used for quantification. Statistical analysis: one-way ANOVA followed by Tukey's multiple comparisons test (A), two-way ANOVA followed by Tukey's multiple comparisons test (C and E). Error bars indicate mean ± s.e.m. and $*p < 0.05$, $**p < 0.01$, $***p < 0.001$.
