# Supplementary material for: The Parkinson’s disease DJ-1/PARK7 gene controls peripheral neuronal excitability and painful neuropathy
Source: Brain. 2024 Nov 2;148(5):1639–51. doi: 10.1093/brain/awae341 (PMC12073980; doi:10.1093/brain/awae341)
Supplement: awae341_Supplementary_Data [file awae341_supplementary_data.zip › brain-2024-01276-File008.pdf]

Supplementary Table 1. Primer sequences for qPCR

Target gene (Product length)	Primers	Genbank No.
Gapdh (95bp)	AGGTCGGTGTGAACGGATTTG<br>GGGGTCGTTGATGGCAACA	NM_001289726
Trpv1 (142bp)	CCGGCTTTTTGGGAAGGGT<br>GAGACAGGTAGGTCCATCCAC	NM_001001445.2
Trpa1 (176bp)	CTACTGGCTTTTGGCCTCAG<br>CCAAAGGTCAGGACTGGGTA	NM_177781.5
Trpm8 (132bp)	ACAGACGTGTCCTACAGTGAC<br>GCTCTGGGCATAACCACACTT	NM_134252
P2rx3 (107bp)	AAAGCTGGACCATTGGGATCA<br>CGTGTCCCGCACTTGGTAG	NM_145526
Th (179bp)	GTCTCAGAGCAGGATACCAAGC<br>CTCTCCTCGAATACCACAGCC	NM_009377.2
Gfap (126bp)	CGGAGACGCATCACCTCTG<br>AGGGAGTGGAGGAGTCATTCG	NM_001131020
Il6 (131bp)	ACAACCACGGCCTTCCCTAC<br>TCCACGATTTCCCAGAGAACA	NM_031168.2
Tnfα (131bp)	CCCCAAAGGGATGAGAAGTT<br>CACTTGGTGGTTTGCTACGA	NM_013693
Iba-1 (144bp)	ATCAACAAGCAATTCCTCGATGA<br>CAGCATTCGCTTCAAGGACATA	NM_001361501.2
Nfe2l2 (153bp)	TAGATGACCATGAGTCGCTTGC<br>GCCAAACTTGCTCCATGTCC	NM_010902.5
Sod1 (131bp)	GGCTTCTCGTCTTGCTCTC<br>TGCTCGAAGTGGATGGTTC	NM_011434.2
Nqo1 (144bp)	AGGCGTCCTTCCTTATATGCTA<br>AGGATGGGAGGTACTCGAATC	NM_008706.5
Ucp4 (123bp)	CCACCCACGGCTTATCCAG<br>ACAAAAGTCCCCTTCCTTGTTT	NM_028711.4
Uchl1 (235bp)	GATGCTGAACAAAGTGTTGGC<br>GGAGTTTCCGATGGTCTGCTT	NM_011670
Sod3 (142bp)	TGCTGGCCTGAACTTCAC<br>ACTCCCCTGGATTTGACATG	NM_011435
Gpx1 (150bp)	GAGTCCCAACATCTCCAGTATG<br>GCGACATTCTCAATGAGCAG	NM_001329528.1
