# Supplementary material for: The Parkinson’s disease DJ-1/PARK7 gene controls peripheral neuronal excitability and painful neuropathy
Source: Brain. 2024 Nov 2;148(5):1639–51. doi: 10.1093/brain/awae341 (PMC12073980; doi:10.1093/brain/awae341)
Supplement: awae341_Supplementary_Data [file awae341_supplementary_data.zip › brain-2024-01276-File011.pdf]

Materials and methods

Animal models of peripheral neuropathy

The chemotherapeutic drugs paclitaxel and vincristine were used to induced painful peripheral neuropathies, as previously described. [1] For paclitaxel, 6 mg/mL stock of paclitaxel was diluted with Cremophor EL and 95% dehydrated ethanol (1:1 ratio) and administered intraperitoneally at a dosage of 2 mg/kg in saline, every other day for a total of 2 injections. Control animals received an equivalent volume of a vehicle containing proportional amounts of Cremophor EL and 95% dehydrated ethanol, also diluted in saline. For vincristine, vincristine sulfate was dissolved in saline, and mice were given a single dose of 200 μg/kg of vincristine via intraperitoneal injection. Control animals received an equivalent volume of saline solution.

Animal model of methylglyoxal-induced nociception

Methylglyoxal was diluted in PBS for intraplantar injection (30 μg) based on previous publications. [2,3] Immediately following the injection of PBS or methylglyoxal into the plantar surface of the hind paw, animals were placed in an acrylic observation box positioned on a transparent platform. A mirror was situated beneath the box to facilitate the observation of licking, lifting, and flinching behaviours. The nocifensive responses of the injected hind paw were monitored and recorded for a duration of 5 minutes. Mechanical sensitivity was also assessed in these mice by von Frey test, as reported below.

Behavioural testing

Open field test

Spontaneous activity was assessed by individually placing mice in the middle of an open field chamber (40cm x 40cm x 40cm) and allowing them to explore freely for 15 minutes. Movement trajectories were recorded using a video camera and walking distance was analysed offline using ANY-maze (Stoelting Co.).

Adhesive Removal (sticky-tape) test

Sensorimotor coordination was assessed by placing mice in clear acrylic boxes for a minimum of 30 minutes to acclimatise. We then applied a 9mm diameter circular adhesive tape (Diversified Biotech) to each animal's hind paw, ensuring equal pressure. We calculated the time it took for the mice to bite, lick, or attempt to remove the tape using previously described methods. [4]

Rotarod test

Forced motor activity was tested by measuring the performance on an accelerating rotarod (IITC Life Science) with the rod programmed to accelerate 5 to 30 r.p.m. over 3 minutes. Each mouse was tested three times and the falling latency was recorded and averaged.

Von Frey test

Static mechanical sensitivity thresholds were assessed using a series of calibrated von Frey filaments (0.02, 0.07, 0.16, 0.4, 0.6, 1.0, and 1.4 g, Stoelting Co.). Animals were acclimatised in a plastic cage with a wire mesh floor and then tested with von Frey filaments (starting with 0.6 g). Each filament was applied for 2-3 s to the glabrous skin on the hind paw, and a positive response was determined by the brisk withdrawal or shaking of the hindpaw. Whenever a positive response occurred, the next lower hair was applied, and whenever a negative response occurred, the next higher hair was applied. The testing consisted of six stimuli, and the pattern of response was converted to a 50% mechanical sensitivity threshold using the up–down method, as we previously described. [5]

Brush test

Dynamic mechanical sensitivity was measured using a paintbrush stimulating the lateral side of the hind paw from heel to toe. Responses were scored on a scale of 0 to 3, constituting an allodynia score. Here, 0 indicates no response or occasional walking away; 1 signifies lifting the stimulated paw towards the body; 2 denotes strong lateral lifting of the paw above the body level; and 3 represents shaking or licking the paw. To determine each animal's average score, this test was performed three times on each paw at 10-second intervals.

Hargreaves test

Heat sensitivity was measured in mice using the Plantar Test apparatus (IITC Life Science) and assessing paw withdrawal latency in response to heat stimulation, according to the Hargreaves method. [6] Animals were first acclimatised in plastic observation boxes for 20 min. Then, the mid-plantar surface of the mouse hindpaw was exposed to a radiant heat source through a glass floor until paw withdrawal. The intensity of the heat was adjusted to produce a baseline of about 15 s in naïve mice, with a maximum cut-off of 20 s. Each mouse was tested three times and averaged.

Tail immersion test

Heat sensitivity was also measured in mice using the tail-flick test involving the immersion of an animal's tail in a heat source. The water bath should be maintained at constant temperatures of 48, 50, or 52 °C. The distal 2 cm of the mouse's tail was immersed in the water bath and its latency to flick was then measured.

Dry ice test

Cold sensitivity was assessed in mice acclimatised to the Plantar Test apparatus (IITC Life Science) for 20 min before cold stimulation with dry ice, according to a previously described protocol. [7] Briefly, a 10 ml syringe, sectioned above the Luer lock and tightly packed with finely crushed dry ice, was pressed firmly on the bottom of the tempered glass directly below the hindpaw. The paw withdrawal latency of the mice was measured with a stopwatch. A standard baseline and a cut-off of 20 s were observed in naïve mice. All mice were tested three times and averaged.

Cold plate test

Cold sensitivity was also assessed by placing mice on a cooled metal surface with a transparent beaker and allowing them to acclimatise to the testing apparatus at room temperature for 30 min. The latency was recorded from placement to a nociceptive behaviour (shaking or licking of a hind paw, jumping). Temperatures of 10, 0, and -10°C were applied with cut-off times of 5 min.

Acetone evaporation test

Cold sensitivity was also assessed by placing mice on a mesh rack within a plastic cage and allowing them to acclimatise for 30 min. Acetone (50 µl) is then applied to the mid-plantar area of

the hind paw. The time spent flicking or licking the paw for the next 120 sec was then measured. The initial 10 sec of activity were disregarded, as this is a response to the direct droplet application.

Mouse DRG dissection and culture

DRG cultures were prepared as previously described. [8] In brief, adult mice aged 6–12 weeks were euthanised using isoflurane and DRGs were harvested from all segments of the spinal cord. After enzymatic dissociation in papain (cat# P3125, MilliporeSigma) and collagenase (cat# C6885, Millipore Sigma), DRGs were triturated with decreasing pipette tips (1 ml, 200 µl) and then cultured the cells in Dulbecco's Minimal Essential Medium (DMEM, cat# 15017CV, Corning Inc.) supplemented with 10% foetal bovine serum (FBS) and 1% penicillin/streptomycin (P/S). Cells were seeded in eight-well plates coated with Geltrex (cat# A1569601, Thermo Fisher Scientific) or in laminin-coated coverslips. Cultures were maintained at 37 °C with 5% carbon dioxide for at least 24 h prior to each experiment.

Measuring Oxidative Stress and Neurite Outgrowth in DRG Cultures

DRG cultures were prepared as described above and then treated with either H2O2 (100 µM) or rotenone (2.5 µM) for 24 hours. Oxidative stress was assessed using dihydroethidium (DHE, cat# D7008, MilliporeSigma) and 4-hydroxynonenal (4-HNE) levels. Briefly, a 3 µM DHE solution was applied to cultured DRG neurones for 15 min and then rinsed before analysis, as we previously described. [5] Analysis of 4-HNE was performed by fixation of cultured DRG neurones and then incubation with 4-HNE primary antibody (rabbit, 1:1000, cat# ab46545, Abcam) overnight followed by 1 h incubation with the secondary antibody Alexa Fluor 546 (cat# A10040, Thermo Fisher Scientific). Images were acquired using the Keyence BZ-X800 microscope, and DHE and 4-HNE intensity levels analysed using NIH Image J software. For quantification, signal intensities were measured by a blinded observer from at least 3 different culture fields per each sample/condition. Neurite outgrowth was assessed by immunofluorescence 72 hours after plating the cultured neurones, as previously described. [9] Briefly, cultures were post-fixed for 20 minutes, and the blocked with the BlockAid™ blocking solution (cat# B10710, Thermo Fisher Scientific) in PBS for 30 minutes. To visualize neurites, we incubated our cultures overnight at 4°C with the β-tubulin antibody (mouse, 1:1000, cat# ab78078, Abcam). The following day, the tissue sections were washed with PBS then incubated for 1 h at room temperature with the secondary antibody

Alexa Fluor 488 (1:500, cat# R37114, Thermo Fisher Scientific). Cultures were then observed, and images captured using the Keyence BZ-X800 microscope. ImageJ software (National Institutes of Health) was used to quantify the average neurite length. The analysis included three images from each of four distinct wells per group. The average neurite length was determined by dividing the total neurite outgrowth by the number of neuron bodies in each image.

Immunofluorescence

Mice were deeply anaesthetised with isoflurane and transcardially perfused with PBS followed by 4% paraformaldehyde (PAF). Lumbar DRG and spinal cord tissues were isolated and post-fixed in PAF for 1–2 h before incubation overnight in a 30% sucrose solution. Tissues were then embedded in OCT medium (Tissue-Tek) and cryosectioned at a thickness of 12 µm. Tissue sections were blocked with BlockAidTM blocking solution (cat# B10710, Thermo Fisher Scientific) or 1% Bovine serum albumin in PBS for 30 minutes and incubated overnight at 4°C with the following primary antibodies: DJ-1 (rabbit, 1:1000, cat# NB300-270, Novus), CGRP (1:500, cat# ab36001, Abcam), NF200 (1:1000, cat# MAB5266, MilliporeSigma), 4-HNE (rabbit, 1:1000, cat# ab46545, Abcam). The following day, the tissue sections were washed with PBS then incubated for 1 h at room temperature with the appropriate following secondary antibodies: Alexa Fluor 546 (1:500, cat# A10040, Thermo Fisher Scientific) and Alexa Fluor 488 (1:500, cat# R37114, Thermo Fisher Scientific), occasionally followed by isolectin B4 (cat# I32450, Thermo Fisher Scientific) or DAPI (cat# D1306, Thermo Fisher Scientific) staining. Images were acquired using the Keyence BZ-X800 microscope. A blinded observer quantified signal intensity using at least three sections per mouse and three to five mice per group. This was done using CellSens (Olympus) or NIH Image J software.

Quantification of intraepidermal nerve fibers (IENF)

Biopsies from the mouse plantar surface were taken, fixed with 4% PFA for 2 h, and cryoprotected in 30% sucrose. Tissues were sliced into 40-µm sections and prepared for staining in PBS solution. Sections were blocked for 1 h at room temperature in 1% BSA solution and incubated with primary antibodies against the pan-neuronal marker PGP9.5 (rabbit, 1:500; cat# Z511601-2; Agilent) overnight, followed by Alexa Fluor 546 (1:500, cat# A10040, Thermo Fisher Scientific) for 1 h. DAPI (cat# D1306, Thermo Fisher Scientific) was used for counterstaining. Images were acquired

using the Keyence BZ-X800 microscope. A blinded observer quantified the density of the IENFs, as previously described. [5]

In situ hybridization (i.e. RNAscope)

Mice were deeply anaesthetised with isoflurane and transcardially perfused with PBS followed by 4% paraformaldehyde (PAF). Lumbar DRGs were isolated and post-fixed in PAF for 1–2 h before incubation overnight in a 30% sucrose solution. Tissues were then embedded in OCT medium (Tissue-Tek) and cryosectioned at a thickness of 12 μm. RNAscope was performed following the manufacturer's instructions using the RNAscope Multiplex Fluorescent Reagent Kit v2 (cat# 323110, Advanced Cell Diagnostics). Probe against the mouse TRPA1 mRNA (cat# 400211, Advanced Cell Diagnostics) was used for RNAscope and with immunofluorescence for DJ-1, which was carried out as described above. Images were acquired using the Keyence BZ-X810 microscope with at least 3 sections from 3 animals included for data analysis. Cells with more than 5 puncta per cell were classified as positive for TRPA1 mRNA expression.

Quantitative Real-Time RT-PCR (qPCR)

DRG tissues were rapidly removed from terminally anaesthetised mice. Total RNA was extracted from these samples using the Direct-zol RNA MiniPrep kit (cat# R2053, Zymo Research), and its amount and purity were assessed by SimpliNano UV–Vis Spectrophotometer (General Electric). Total RNA was converted into cDNA using a high-capacity cDNA reverse transcription kit (cat# 4368814, Thermo Fisher Scientific). Specific primers used in this study were obtained from PrimerBank (https://pga.mgh.harvard.edu/primerbank/), and their sequences are shown in Supplementary Table 1. qPCR was performed on the QuantStudio 3 Real-Time PCR System (Thermo Fisher Scientific) using PowerUp SYBR Green Master Mix (cat# A25741, Thermo Fisher Scientific). All samples were normalised by Gapdh expression. Relative transcriptional expression ratios per condition were calculated as previously described. [5]

Reverse-transcription polymerase chain reaction (RT-PCR)

cDNA was synthesised in the same way as qPCR. Samples were diluted 2:100 and used as template for PCR experiments. The following primer pairs were used: mouse DJ-1 (forward, 5'-CAAAGGAGCAGAGGAGATGG-3'; reverse,5'-CCTTAGCCAGTGGGTGTGTT-3'), mouse

Gapdh (forward, 5'-TGAAGGTCGGTGTGAACGAATT-3'; reverse, 5' GCTTTCTCCATGGTGGTGAAGA-3'), human DJ-1 (forward, 5' GGAGACGGTCATCCCTGTAG-3'; reverse, 5'-TTCACAGCAGCAGACTCAGA -3'), human Gapdh (forward, 5'- ACCCAGAAGACTGTGGATGG-3'; reverse, 5'-TTCTAGACGGCAGGTCAGGT-3'). RT-PCR analysis was performed using human DRG tissues from a deidentified organ donor and approved by the University of Cincinnati Institutional Review Boards.

Western blot

Protein samples were prepared as previously described. [10] Briefly, we mixed them with RIPA lysis buffer (cat# 20-188, MilliporeSigma), then homogenised them with a glass grinder to extract the total protein. The protein concentrations were determined using the Qubit protein assay (cat# Q33211, Thermo Fisher Scientific). We loaded 20 micrograms of protein into each lane, then separated it using a 10% SDS-PAGE gel and transferred the protein to a PVDF membrane. After blocking the Western blotting membranes with SuperBlock™ Blocking Buffer for 10 minutes, we left them to incubate overnight at 4°C with oxidised DJ-1 antibody (rabbit, 1:1000; Abcam; cat# ab169520). After that, the membranes were incubated with a rabbit secondary antibody (1:2000; Cell Signaling, cat# 7074) for 1 hr and developed in SuperSignal™ West Dura Extended Duration Substrate (cat# 34076, Thermo Fisher Scientific). The membranes were scanned with iBright FL1000 (Invitrogen) and specific bands were identified based on their apparent molecular sizes. We used ImageJ software (National Institutes of Health) to calculate the ratio of band intensities of protein normalised to GAPDH (rabbit, 1:2000, cat# NB100-56875, Novus).

Calcium imaging

Cultured DRG neurones were prepared as described above and Fura-2 AM-based calcium imaging experiments performed, as previously described76. Briefly, DRG neurones prepared were loaded with Fura-2 AM (2 μM) for 40 min at 37°C in DMEM. Extracellular solution contained (in mM) 140 NaCl, 5 KCl, 10 HEPES, 1 CaCl2, 2 MgCl2 and 10 D-(+)-glucose, pH 7.4. Acquired images were displayed as the ratio of 340 nm to 380 nm. Cells were illuminated with lamp and were excited by Lambda DG-4 (Shutter Instrument), and identified as neurones by eliciting depolarisation with high potassium solution (50 mM) at the end of each experiment. The

fluorescence 340/480 nm ratio was measured by digital video microfluorometry with camera (optiMOS, QImaging) coupled to the inverted BX51WI microscope (Olympus) and a computer with software (Slidebook 6, 3i, Intelligent Imaging Innovations). All drugs were applied via bath perfusion at a flow rate of 1 mL/min.

Human DRG dissection and culture

Human DRG tissues were rapidly dissected from the nerve roots, minced in Ca2+-free Hank's balanced salt solution (Gibco), and digested at 37 °C in a humidified CO2 incubator for 180 min with collagenase type II (390 units/mg; 12 mg/ml) and dispase II (1 unit/mg, 20 mg/ml) in PBS with 200 μM sodium pyruvate and 10 mM HEPES. The pH was adjusted to 7.4 with NaOH. The sample was then centrifuged for 5 min at 400× g to pellet the ganglia and the collagenase type II and dispase II solution was carefully removed. Five mL of prewarmed DMEM supplemented with 10% FBS and 1% penicillin/streptomycin was added, and the cells were mechanically dissociated. The solution was then filtered through a 100-μm nylon mesh and centrifuged for 5 min (500× g). The DRG cell pellet was resuspended and plated on 0.1 mg/mL Corning Cell-Tak-coated glass coverslips. The DRG cultures were grown in neurobasal medium supplemented with 10% FBS, 2% B-27 supplement, 1% N2 supplement, and 1% P/S. After five days of culture, human DRG neurones were used for whole-cell patch-clamp recording.

Whole-cell Patch-clamp Recordings

Whole-cell patch-clamp recordings were conducted using mouse and human cultured neurones prepared as described in the method above. Cultured neurones were maintained at 25°C and recording performed with MPC-200 manipulators (Sutter Instrument) and a Multiclamp 700B amplifier (Molecular Devices). The patch pipettes were pulled from borosilicate capillaries (Sutter Instruments). The resistance of the pipette was 5-8 MΩ. The recording chamber (volume 300 μL) was continuously perfused (2-3 mL/min). The pipette solution for voltage-clamp and current-clamp experiments contained the following (in mM): 126 K-gluconate, 10 EGTA, 1 MgCl2, 2 NaATP, 0.1 Na3GTP, and 10 HEPES; the pH was adjusted to 7.3 with KOH. Data were low-pass filtered at 2 kHz and sampled at 10 kHz. The bath solution contained the following (in mM), 140 NaCl, 5 KCl, 2 CaCl2, 1 MgCl2, 10 HEPES and 10 glucose; the pH was adjusted to 7.4 with NaOH. Voltage clamp recordings were performed at a holding potential of -60 mV. In current

clamp recordings, the action potentials were evoked by current injection steps. The resting membrane potential (RMP) was measured without current injection. The pClamp10 (Molecular Devices) software was used during experiments and analysis.

1. Alessandri-Haber N, Dina OA, Joseph EK, Reichling DB, Levine JD. Interaction of transient receptor potential vanilloid 4, integrin, and SRC tyrosine kinase in mechanical hyperalgesia. *J Neurosci*. Jan 30 2008;28(5):1046-57. doi:10.1523/jneurosci.4497-07.2008
2. Bierhaus A, Fleming T, Stoyanov S*, et al*. Methylglyoxal modification of Nav1.8 facilitates nociceptive neuron firing and causes hyperalgesia in diabetic neuropathy. *Nat Med*. Jun 2012;18(6):926-33. doi:10.1038/nm.2750
3. Yousuf MS, Sahn JJ, Yang H*, et al*. Highly specific σ(2)R/TMEM97 ligand FEM-1689 alleviates neuropathic pain and inhibits the integrated stress response. *Proc Natl Acad Sci U S A*. Dec 26 2023;120(52):e2306090120. doi:10.1073/pnas.2306090120
4. Bouet V, Boulouard M, Toutain J*, et al*. The adhesive removal test: a sensitive method to assess sensorimotor deficits in mice. *Nat Protoc*. 2009;4(10):1560-4. doi:10.1038/nprot.2009.125
5. Tonello R, Lee SH, Berta T. Monoclonal Antibody Targeting the Matrix Metalloproteinase 9 Prevents and Reverses Paclitaxel-Induced Peripheral Neuropathy in Mice. *J Pain*. May 2019;20(5):515-527. doi:10.1016/j.jpain.2018.11.003
6. Hargreaves K, Dubner R, Brown F, Flores C, Joris J. A new and sensitive method for measuring thermal nociception in cutaneous hyperalgesia. *Pain*. Jan 1988;32(1):77-88. doi:10.1016/0304-3959(88)90026-7
7. Brenner DS, Golden JP, Gereau RWt. A novel behavioral assay for measuring cold sensation in mice. *PLoS One*. 2012;7(6):e39765. doi:10.1371/journal.pone.0039765
8. Lee SH, Cho PS, Tonello R*, et al*. Peripheral serotonin receptor 2B and transient receptor potential channel 4 mediate pruritus to serotonergic antidepressants in mice. *J Allergy Clin Immunol*. Oct 2018;142(4):1349-1352.e16. doi:10.1016/j.jaci.2018.05.031
9. Mersman B, Zaidi W, Syed NI, Xu F. Taurine Promotes Neurite Outgrowth and Synapse Development of Both Vertebrate and Invertebrate Central Neurons. *Front Synaptic Neurosci*. 2020;12:29. doi:10.3389/fnsyn.2020.00029

10. Lee SH, Tonello R, Choi Y, Jung SJ, Berta T. Sensory Neuron–Expressed TRPC4 Is a Target for the Relief of Psoriasiform Itch and Skin Inflammation in Mice. *Journal of Investigative Dermatology*. 2020;140(11):2221-2229.e6. doi:10.1016/j.jid.2020.03.959
